# Mineral, Nutritional, and Phytochemical Composition and Baking Properties of Teff and Watermelon Seed Flours

**DOI:** 10.3390/molecules28073255

**Published:** 2023-04-05

**Authors:** Anna Jaroszewska, Dariusz Jedrejek, Magdalena Sobolewska, Iwona Kowalska, Małgorzata Dzięcioł

**Affiliations:** 1 Department of Agroengineering, Faculty of Environmental Management and Agriculture, West Pomeranian University of Technology in Szczecin, Pawła VI Street, 71-459 Szczecin, Poland; anna.jaroszewska@zut.edu.pl (A.J.); magdalena.sobolewska@zut.edu.pl (M.S.); 2 Department of Biochemistry and Crop Quality, Institute of Soil Science and Plant Cultivation—State Research Institute, Czartoryskich 8 Street, 24-100 Puławy, Poland; ikowalska@iung.pulawy.pl; 3 Department of Chemical Organic Technology and Polymeric Materials, Faculty of Chemical Technology and Engineering, West Pomeranian University of Technology in Szczecin, Piastów Ave. 42, 71-065 Szczecin, Poland; malgorzata.dzieciol@zut.edu.pl

**Keywords:** *Eragrostis tef*, *Citrullus lanatus*, farinographic analysis, minerals, nutrients, flavonoids, melatonin, antioxidants

## Abstract

Demonstrated limitations in the mineral and nutritional composition of refined flours have led to calls for the possibility of enriching them with health-promoting supplements, such as high-value non-cereal seeds. Teff and watermelon seeds have been found suitable for the production of gluten-free flour, but so far, their potential to enrich conventional baking flours has not been comprehensively studied. Hence, the present study aimed at farinographic evaluation of dough based on refined wheat flour with additions of whole white teff (TF) and watermelon seed (WSF) and pomace (DWSF) flours (tested levels 10%, 20%, and 30%), as well as possibly extensive chemical characterization of the plant material tested, including LC-MS/MS, GC-MS, total phenolics, flavonoids, melatonin, and antioxidant potential. Most of the rheological traits were improved in the flour mixtures compared to the base white flour: development time and quality number (above 1.6-fold increase), softening and stability time (up to 1.3-fold change), and water absorption (up to 6%). Overall, the best results were achieved after the addition of watermelon seed pomace. The DWSF material was characterized by the highest levels of P, Mg, Na (7.5, 1.7, 0.4 g/kg, respectively), and Fe and Zn (124 and 27 mg/kg), while TF was the richest in Ca (0.9 g/kg) and Mn (43 mg/kg). Protein and fat levels were significantly higher in watermelon seeds compared to teff (about double and up to 10-fold, respectively). Phytochemical analyses highlighted the abundance of phenolics, especially flavones, in TF, WSF and DWSF flours (244, 93, and 721 mg/kg, respectively). However, the value of total polyphenols was low in all materials (<2 mg GAE/g), which also correlates with the low antioxidant potential of the samples. Watermelon seed pomace was characterized by significantly higher melatonin concentration (60 µg/kg) than teff (3.5 µg/kg). This study provides new information on the chemical composition and application opportunities of teff and watermelon seeds.

## 1. Introduction

A fundamental role in the nutrition of Western societies is played by gluten cereal products; therefore, the composition and content of nutrients and other beneficial phytochemicals in grain and flour are of great importance. However, the vast majority of bakery products available and consumed are made from white flour [1,2], which is obtained from grinding refined grain (after dehulling). Various studies report that bran fraction contains large amounts of valuable minerals (e.g., Zn, Fe, and Se), fiber, and phenolics/antioxidants, so the refining process adversely affects the nutritional and health-associated traits of grain. It has been shown that replacing white-flour bakery products with those made from whole grain can reduce the risk of common ailments, such as obesity, type 2 diabetes, and cardiovascular diseases [3,4,5]. In addition, attention has been drawn for some time to the deteriorating nutritional and health-promoting quality (depletion in micronutrients and bioactive substances) of cereal grains from intensive cultivation, often being monocultures, and from newly introduced crop varieties focused mainly on yield, which ultimately has negative consequences for the human diet [6,7].

The staple flours used in conventional baking are derived from wheat or rye grain, in which the gluten protein fraction is responsible for the formation of a viscoelastic dough and its rise during baking. Due to several factors (the incidence of food allergies and celiac disease, dietary trends, and the growing interest in functional foods), other grain and non-grain flours are beginning to play an increasingly important role in baking [8]. Unfortunately, the elimination of gluten from the baking process results in a decline in the quality of bakery products, due to deterioration in texture, sensory characteristics and overall quality [9]. The problem does not occur when other (gluten-free) flours are merely additives to the base gluten flours; however, the development of new product formulations based solely on gluten-free flours, which would be fully accepted by consumers, remains a challenge for the food industry.

Recently, there has been growing interest in the utilization of other high-quality grains (e.g., oats, quinoa, amaranth, teff, and pumpkin and legume seeds) to enrich wheat flours in the production of cereal-based foods [10]. Various studies have demonstrated that the abovementioned gluten-free seeds are characterized by favorable parameters for human dietary needs, including increased content of valuable minerals, fiber, and nutrients (protein, fatty acids, and vitamins), which are far superior to the composition of cereal bakery flours, both refined and whole-grain [2,11,12,13,14]. Teff (*Eragrostis tef*) is a grass species native to Ethiopia, with very fine seeds and remarkable drought tolerance. This cereal is mainly grown in Ethiopia, with an annual yield of about 5 million tons [15]. In its homeland, teff grain is mainly used for the production of flatbread. Previous studies have shown that *E. tef* seeds are abundant in Ca, Fe and Zn, while the protein content is at a similar level as in wheat [16,17]. Watermelon (*Citrullus lanatus*) is an annual cucurbitaceous plant, widely grown in many countries with a sunny and humid climate. Its annual production is about 100 million tons, and the main producers are China (60% of global production), Turkey, India and Brazil [18]. Seeds were until recently treated mainly as bio-waste, but are now increasingly being used for edible oil, flour, or snacks. *C. lanatus* seeds have been shown to be highly abundant in P, K, Mg, Mn, and Zn [19], and their protein and lipid content have been found at a much higher level than in other grains, including cereal (wheat, whole wheat, oat, maize and teff) and non-cereal materials (buckwheat and quinoa) [17,20].

In addition to providing nutrients and minerals, plant foods are a source of natural molecules with pro-health properties, such as antioxidant, anti-inflammatory, antimicrobial, immune-boosting, and aiding in digestion. Among the most common specialized metabolites found in plant materials are phenolics, a group of substances with an overabundance of chemical structures (including phenolic acids and flavonoids) that are primarily attributed to antioxidant and anti-inflammatory effects [21,22,23]. Given the gaps in the recognition of the qualitative and quantitative phytochemical composition of many raw materials and plant products, as well as the newly described biological properties of natural compounds, phytochemical profiling of plants is still one of the goals of many scientific studies.

Dough rheological evaluation allows the flour to be tested under production-like conditions in a bakery, thus enabling a more complete determination of its quality and machinability than basic parameters such as carbohydrate and protein content [24,25]. Teff and watermelon seeds have been found suitable for the production of gluten-free flour [19,26,27], but so far, the effects of their addition to wheat flour on dough rheology and their potential to supplement refined flours with nutrients and bioactive phytochemicals have not been comprehensively studied. Thus, the purpose of our study was to address the following issues:

- determining the nutritional and health benefits of using teff and watermelon seed flour (including whole seed and pomace flour) to enrich white flour by analyzing the mineral, nutritional, phytochemical, and antioxidant composition;
- determining the suitability of teff and watermelon seed flours in combination with refined flour for baking purposes by analyzing the rheological parameters of the dough based on mixtures of flours.

## 2. Results and Discussion

### 2.1. Mineral Content

Minerals are essential for the proper functioning of every organism on earth, as they serve as cofactors for many physiological and metabolic processes. In humans, they are part of bones, teeth, blood, and hair. Mineral contents of teff (TF) and watermelon seed flours (pomace-DWSF and seed-WSF) are presented in Table 1, and as can be seen, the material differed significantly in the concentrations of most of the analyzed macro- and microelements. Watermelon seed pomace presented the highest levels of P, Mg, Na, Fe, Zn, and Mo, which were 10% (Mg and Zn) to 300% (Na and Fe) lower in other samples. In contrast, teff contained the highest Ca and Mn, which were about a third lower in watermelon seed materials. Potassium levels in watermelon were about twice those in teff. No copper or toxic metals (Cd and Pb) were observed in any of the flour materials (Table 1).

Several works exist on the mineral content of both ground teff and watermelon seeds; however, the reported elemental values are characterized by large ranges of variability, as exemplified by the Fe level in teff (47–363 mg/kg, Table S1). It has been previously shown that *E. tef* seeds are highly abundant in calcium, iron, phosphorus, and zinc, with concentrations up to several times those determined in other cereals—barley, corn, rice, and wheat [16,17]. Overall, the levels of Ca, Mg, Zn, and Mn in teff measured in this study are consistent with the literature. On the other hand, the P concentration is above the highest known value (4.8 g/kg, Table S1). As for iron, our analysis indicates that its level (60 mg/kg) is significantly lower than the reported maximum values (>300 mg/kg, Table S1). *C. lanatus* seeds have been demonstrated to be abundant in many minerals, including P, K, Ca, Mg, Zn, Cu, and Mn [19], and obtained results confirm this (except for Cu—not detected, and Ca—value below the reported minimum; Table S1).

Due to their widespread consumption and mineral composition, wheat-based foods are considered an important source of many macro- (Ca, Mg, K, and P) and microelements (Fe, Cu, Mn, and Zn) in the diet [1]. In this respect, wheat is far superior to the other two mass-produced grains—maize and rice [17]. On the other hand, it is known that the total grain purification process has a negative impact on the final mineral content of the flour: a comparison of white and whole-grain wheat flour shows that the levels of the aforementioned minerals fall about two- to threefold as a result of refining [1,17]. In some countries, this problem is addressed through mandatory fortification of refined flours with some (but not all) of the minerals (e.g., Fe) to compensate for processing losses.

Previous results and ours confirm that teff and watermelon seeds have clearly higher levels of several important elements (Ca, Fe, Mn and Zn in teff and P, K, Mg, Fe, and Zn in watermelon seeds) than the three most commonly grown cereals (wheat, maize and rice); therefore, these materials have high potential to enrich the mineral composition of white baking flours.

### 2.2. Protein and Lipid Content

The content and quality of protein in the grain are essential parameters in assessing its baking value. The total protein measured in test flours was in the range of 11.7–25.2% of dry mass (DM) (Table 1), and its level in watermelon material (DWSF and WSF) was about double that of teff. Previous reports place the protein concentration in *E. tef* flour at a similar level as wheat, spelt, and buckwheat (≥10%), but superior to oat, rice, maize, and sorghum (≤8%) [8,17], while the values determined in *C. lanatus* seeds (>20%) are well above typical grains [28]. Thus, obtained results confirm previous works.

The determined lipid content of test flours ranged from 2.9% to 29.6% DM, and it was several times higher in watermelon samples than in teff (Table 1). As above, the obtained results confirm previous works. The fat level in teff grain has been previously described (3–4.5%) as superior to wheat, rice, and maize (two- to fivefold), but much lower than in oats (half) [17]. While watermelon seeds are much richer in lipids than cereal or other gluten-free seeds [20], the reported values cover a fairly large range of 19–50% (Table S1). Due to the high proportion of polyunsaturated fatty acids (linoleic acid (C18:2) content > 60%), *C. lanatus* seeds are sometimes used to extract food-grade oil [20,29]. As the resulting seed pomace is still characterized by the presence of valuable nutrients and bioactive constituents (such as minerals, proteins, and phytochemicals), research is undertaken for their further use, e.g., as a supplement to refined flours.

### 2.3. Phytochemical Screening

#### 2.3.1. UHPLC-PDA-MS/MS Analysis

A total of 27 metabolites were characterized in aqueous methanolic extracts of three flour materials (including 13 metabolites in teff and 17 metabolites in watermelon seed material), and the list of compounds is presented in Table 2. In addition, the LC-PDA-MS chromatograms are presented in Figures S3–S5. The majority of the chromatographic signals were identified as flavonoids (16 compounds), mainly luteolin or apigenin derivatives. As suspected, the registered metabolic profiles were characterized by species specificity, but with several compounds present in both types of plant material (e.g., *C*-hexoside of luteolin and apigenin). Flavonoids observed in *E. tef* flour occurred only as glycoside derivatives, while flavone aglycones predominated in *C. lanatus* material. Both watermelon samples (seed and pomace) had highly similar qualitative metabolic profiles (Figures S4 and S5) but differed in the intensity of individual constituents (pomace was dominated by luteolin, chrysoeriol, and apigenin, while seeds were abundant in compound 22 (*m*/*z* 337, unidentified), luteolin, and luteolin-7-*O*-glucoside) (Table 2).

Some previous publications indicate that the predominant polyphenols of teff seeds are either phenolic acids or catechins and flavonols [30,31,32], but the most complete picture of the phytochemical profile of *E. tef* seeds, actually dominated by luteolin and apigenin derivatives, is presented in a recent paper by Ravisankar et al. [33]. Our analyses, on the one hand, confirm the above results, i.e., flavone derivatives are the main phenolic compounds, but on the other hand, we demonstrate the presence of luteolin derivatives in white teff, which—according to Ravisankar’s thesis—should only be found in brown teff.

Despite the vast reports on the chemical composition of the *C. lanatus* plant [34,35], it is difficult to find detailed phytochemical data on seed material. Available information on phenolic substances covers only the total polyphenol fraction—TPC [36,37]—so the phytochemical profiling performed in this study is one of the first for this material and fills in the knowledge gaps.

Phytochemicals such as phenolic acids and flavonoids occur widely in plants, as one of their primary functions is to protect them against the harmful effects of UV radiation and reactive oxygen species. Sources of these substances in the human diet are mainly vegetables and fruits, but also nuts and cereal seeds, including flour products [17,38]. Phenolics, among them flavone derivatives, are mainly attributed to antioxidant and anti-inflammatory effects, but they have also been tested successfully for other biological properties, such as cardioprotective, antimicrobial and anticancer. Scientific evidence has shown that a diet rich in these compounds can help prevent some chronic ailments, including type 2 diabetes and cardiovascular diseases [6,11,21].

The role of phenolic compounds in the baking process has not yet been further recognized and described. On the other hand, it was established that phenolic acids are quite stable under baking conditions (similar or even higher amounts are recorded in baked products compared to flour), but glycosidic derivatives of flavonoids, which are present in flour (mainly *O*- and *C*-glycosides of flavones), undergo hydrolysis to aglycone forms (glycoside levels dramatically decrease in bakery products while aglycone levels increase) [22,39].

#### 2.3.2. Total Phenolic (TPC) and Flavonoid Content

Test samples differed significantly in total phenolic content (DWSF > WSF > TF); however, the values obtained (<2 mg GAE/g, Table 3) do not indicate that test flours are particularly rich in these phytochemicals. The TPC data of *E. tef* and *C. lanatus* seeds are quite common in the literature, but their range is unsurprisingly very wide in both cases: teff (0.37–1.84 mg GAE/g) and watermelon seeds (0.04–54 mg GAE/g) (Table S3). The large discrepancy in results may be partly due to the variability of the plant material, including that depending on genetic and cultivation factors, but the different variations in the research methodology used should also be considered (Table S3).

Flavone compounds have been found as predominant specialized metabolites of the test flours, so efforts were made to quantify them using the UHPLC-PDA method. The highest level of flavonoids was determined in watermelon seed pomace (720 mg/kg), which was three- and eightfold that of teff and watermelon seed material, respectively (Table 3). The main single flavones in the DWSF sample were luteolin, apigenin *C*-hexoside and chrysoeriol (372, 103 and 101 mg/kg, respectively). A similar pattern was observed in WSF material, but the calculated concentrations were 5- to 10-fold lower. In contrast, teff seeds were richest in luteolin *C*-hexoside and luteolin di-hexoside (91 and 71 mg/kg, respectively) (Table 3). The total flavonoid content of the analyzed white teff sample was much lower (about sevenfold) than the results obtained earlier by Ravisankar et al. [33] (1400–2050 mg/kg). Given the similarity of the methodology used in both works, it can be assumed that the phytochemical composition of teff grain is strongly dependent on origin. In the case of watermelon seeds, these are the first detailed results about the flavonoid profile and quantitative content of individual compounds.

Phenolic compounds in cereal grains are mainly contained in the coats, and thus they are lost, together with all health-promoting effects they bring with them, during the refining of flour. White (refined) wheat flour contains about 100–200 mg/kg of total phenolics (including mainly hydroxycinnamic acids), while their concentration in whole wheat flour is several times higher (three- to fivefold) [17,39]. Flours made from so-called high-value seeds (buckwheat, quinoa, sorghum, teff and cucurbit seeds) have even higher levels of phenolic substances (from 1000 to as much as 4500 mg/kg) than whole-grain cereal flours [17,40], including a higher proportion of flavonoids, which are attributed comparable or even better antioxidant and anti-inflammatory effects than phenolic acids [41]. Therefore, the abovementioned seeds can be a valuable addition to complement the composition of phenolics in refined flours, providing them in the diet as much (or even more) as whole-grain flours.

#### 2.3.3. GC-MS Analysis

The application of GC-MS enabled the identification of 10 medium- and nonpolar compounds in three seed flours, while the identity of several other components could not be matched (Table 4). The analyzed samples were differentiated in terms of their relative quantitative profile (Figures S6–S8). Linoleic and palmitic acids were among the primary components of watermelon seed extract, but they were not detected in the pomace. Most of the described metabolites were attributed to only one flour type: squalene, stigmasterol, isomultiflorenon, and lupeol were detected in watermelon flours, while β-sitosterol and β-amyrin were found only in teff. In contrast, β-tocopherol and (*Z*)-9-octadecenamide were present, albeit at a low level, in all analyzed materials. In general, all identified metabolites have been previously confirmed as constituents of watermelon [35] or teff material [42]. Nevertheless, it should be noted that they belong to bioactive natural products with important biological properties, such as anti-inflammatory, antibacterial, hepatoprotective and cytotoxic [43].

#### 2.3.4. Melatonin (MEL) Content

Melatonin plays an important role in synchronizing sleep–wake timing and control of seasonal rhythmicity in vertebrates. However, MEL is also abundant in the plant kingdom. To date, the compound has been extracted from numerous crops (cereals, fruits, vegetables, and nuts) and plant products (beverages, edible oils, and medical herbs) [44,45]. Melatonin deficiency in humans results in insomnia and circadian rhythm sleep disturbances [46]. The symptoms of the disorder can be overcome by restoring MEL levels in the body, for examples by providing medication or dietary exogenous MEL. As shown in a human study by Sae-Teaw et al. [47], a diet composed of foods rich in melatonin (some tropical fruits) can raise its serum concentration to the physiological level recorded at night (50–200 pg/mL).

It is assumed that MEL presence in plants is a universal feature and variations in its concentration have mainly a genetic basis, but also depend on plant tissue, growth state, and environment [44]. Our work is the first report on melatonin content in *E. tef* and *C. lanatus* seeds. The developed methodology based on pressurized extraction and UHPLC-MS/MS analysis was characterized by good-quality parameters of R^2^, the goodness of fit, limit of detection, reproducibility, and recovery (Table S4). Representative chromatograms of standard and test samples are provided in Figures S9 and S10. As shown in Table 3, the examined material differed significantly in the MEL content, the highest level was recorded in watermelon seed pomace (63 µg/kg), while it was 6 and 18 times lower in WSF and TF samples, respectively. The presence of melatonin in major cereals has been previously confirmed, showing substantial differences (up to several hundred-fold) in both species and varieties within a single species, for example, rice (0.04–2034 µg/kg), corn (1.0–264 µg/kg), and wheat (2.0–125 µg/kg). Our results indicate that watermelon seeds can be considered a rich source of exogenous MEL, being at or above the level of other important plant-based foods, such as barley, cherries, and strawberries [44,45].

### 2.4. Antioxidant Properties of Teff and Watermelon Seed Material

Scientific evidence has shown that the redox imbalance in the body may lead in the long term to the emergence and development of pathophysiological processes such as cancer, diabetes, and cardiovascular and neurodegenerative diseases. Natural antioxidants, such as phenolics present in most fruits and vegetables, support the functioning of the human detoxification system in coping with oxidative stress, either by preventing the formation or inactivating harmful radicals [21,23]. Many different factors and mechanisms for the formation of these highly reactive molecules have been described, for example, transition metal ions (Fe and Cu), which contain unpaired electrons, usually participate in free radical reactions in the body, serving as a substrate for the formation of reactive oxygen species (ROS) [48]. Therefore, substances capable of chelating such metal ions are classified as antioxidants and are also sought after among natural plant substances. It is generally recommended in antioxidant research that several different tests involving different mechanisms of interaction of active substances with radicals (e.g., radical scavenging and chelation of Fe and Cu metal ions) be used. The ability of natural phenolics to inactivate ROS depends on their chemical structure, including the location and number of hydroxyl groups (the presence/modification of catechol moiety is particularly important) [49].

The antioxidant potential of test flours was measured using ABTS and DPPH antiradical assays, while metal ion chelation efficiency was analyzed via FCA assay. Inhibition curves describing the flour extracts and positive controls are presented in Figure S11, and as can be seen, dose-dependent activity was obtained for all samples. The final results are expressed as IC_50_ values (Table 5). The test flour samples differed significantly in their potential to inactivate radicals (DWSF > TF ≥ WSF), and had a differential ability to complex Fe^2+^ ions (WSF > DWSF > TF). Nevertheless, the activity of the samples was relatively low compared to control substances (Trolox or EDTA) in both types of in vitro tests. The poor antioxidant capacity of flour material is likely due to low detected levels of total phenolics (Table 4), which are usually involved in such action of plant samples. In addition, the rather low level of phenolic compounds in the flours studied is also supported by the low overall yield from metabolite extraction (Section 3.5.1). Moreover, relating the values from antioxidant determinations to the quantity of extracts obtained, it turns out that their antioxidant activity is incomparably higher than that of raw plant material (between 10- and 30-fold; Table 5).

Because of the many modifications to the methodology of the colorimetric determinations themselves (e.g., different proportions of reagents) and the methods of calculating and expressing the results obtained (different units, reporting results per extract or raw material), the task of comparing our results with previous work is not easy and must be carried out with some simplification. The antiradical DPPH potential of teff seeds reported by Forsido et al. [50] was about triple that of our analysis, most likely due to the significantly higher (also about threefold) polyphenol content in that material. In addition, comparative studies of extracts from different types of flours show that the material characterized by a higher polyphenol content (whole wheat > corn > rye > wheat) also presented better DPPH radical scavenging effect (IC_50_ values of 5.6, 17.8, 18.1, and 26.4 mg/mL, respectively) [51]. Convergent results (positive correlation between polyphenol concentration and antiradical activity) of extracts from different types of wheat flours (wheat bran and flour types 850 and 500) are described in the work of Sedej et al. (DPPH IC_50_ values of 31.6, 31.6 and 34.2 mg/mL, respectively) [52]. The same work also investigated the ability of wheat flour extracts to chelate Fe^2+^ ions, obtaining identical results for each sample (regardless of polyphenol content), which were at the same time much lower (IC_50_ value of 0.06 mg/mL) than the values obtained in our test [52].

Overall, the current study confirms that both teff and watermelon seeds exhibit antioxidant activity and their addition to baked products can increase their health-promoting benefits.

### 2.5. Farinographic Evaluation of Dough Based on Wheat Flour with Test Supplements

High-quality bakery products can be obtained when the dough is characterized by optimal rheological properties, considering such parameters as water absorption, time of development and stability, and degree of softening. In our set of experiments, teff and watermelon seed flours were added in amounts ranging from 10% to 30% to the base refined flour (wheat flour type 650) and rheological features were evaluated using the Brabender farinograph method. Obtained results are presented in Table 6 and Figure S2.

The water absorption capacity describes flour’s ability to bind water, and this feature significantly affects the dough yield and influences its texture, crumb and rise, and the appearance of the final product. It has been previously established that the optimal water content of bread dough based on wheat flour should be in the range of 55–65%, while reduced hydration (<55%) unfavorably extends the mixing time [53]. In our experiment, the lowest result was obtained for the control WF flour (55.4%, Table 6), and the addition of each test material significantly increased this parameter. The largest increase (up to 3%) was obtained for watermelon seed pomace. Overall, the potential of additives to increase the hydration of baking flour was as follows: DWSF > TF ≥ WSF. Previous studies have indicated that one of the main factors impacting water absorption of the flour is the amount and quality of protein, particularly the gluten fraction. Dough usually contains 40% to 60% water, which corresponds to 0.67–0.85 g/g of flour, and an increase in its proportion is mostly positively correlated with the concentration of protein in flour [28,53]; however, this dependence does not fully agree with the material tested by us.

Dough development time is measured from the moment the water is added to the flour until signs of diminishing consistency appear. In our experiment, this parameter was in the range of 2.6–6.0 min (Table 6), and the three additives positively prolonged obtained values (from 1.3 to 3.4 min) compared to the control sample (WF, 2.6 min). However, surprisingly, the intensity of the observed effect was inversely related to the additive dose applied: 10% (range 4.6–6.0 min) ≥ 20% (range 4.3–5.5 min) ≥ 30% (range 3.9–4.9 min), which could be due to declining gluten cross-linking process and accelerated hydration when the proportion of additive to base flour is increased. The time of stability describes the resistance of dough to the mixing process. According to a previous study, a value above 5 min suggests a dough with a dense structure that can be stirred longer [53]. In our study, this feature ranged from 4.2 to 8.9 min (Table 6) and the obtained results were not clearly in favor of the additives used when compared with the control (6.8 min). Only the supplementation of WF flour with watermelon seed flours (30% DWSF and 10% WSF) significantly increased the stability time (by 1.9 min on average), but on the other hand, the 30% dose of TF and WSF flours significantly reduced obtained values (by 2.5 min on average). For TF and WSF materials a negative dose-effect correlation was observed. Overall, the potential of test supplements to extend dough stability time was as follows: DWSF > WSF > TF. Previous reports have indicated that the development and stability times of dough mainly depend on the protein content and quality of the flour, and thus also on its ability to absorb water [53], but in our case dough hydration was positively correlated (0.67) only with the development time (Table S2).

Softening informs about the durability of dough against mechanical processing, and according to a previous study, values above 70 FU indicate the dough that can hardly withstand long mechanical treatment [54]. For the analyzed dough samples, this parameter was within a wide range of 28.5–71.5 FU (Table 6). The control wheat flour (WF) had a value of 51 FU (which already proves the good durability), and a significant reduction in the degree of softening (by 7–14 FU) was obtained for the entire tested 10–30% range of the DWSF additive, as well as for the lowest 10% concentration of the TF and WSF additives. In turn, the highest applied 30% dose of TF and WSF materials significantly increased softening (by 20 FU on average) compared to the control refined flour. Overall, the potential of supplements tested to reduce dough softening was as follows: DWSF > WSF ≥ TF. Previous research has shown that the degree of dough softening is negatively correlated with the protein content and other rheological parameters of the flour (water absorption, development time, and stability) [53]. Obtained results support these observations (correlation coefficients from −0.38 to −0.89) (Table S2).

Farinograph quality number (FQN) is a way of expressing the shape of the farinograph curve by a number. The FQN index of analyzed dough samples ranged from 60 to 100 mm (Table 6). A significant increase in the quality number (from 10 to 40 mm) compared to the control sample (62 mm) was obtained for the entire tested 10−30% range of watermelon seed pomace flour, as well as for the lower 10−20% doses of teff and watermelon seed flour. The FQN index of flours containing TF and WSF materials was negatively correlated with the applied dose (10% > 20% > 30%); while the opposite trend was observed for the DWSF material.

As shown in Figure S2, the addition of test flours had the strongest effect on dough development time and the quality number (up to 2.3- and 1.6-fold increase, respectively), to a lesser extent on the softening and stability time (both up to 1.3-fold change), while the most subtle alteration was registered for water absorption capacity. The high variability in rheological data between the test flour mixtures can be explained by qualitative and quantitative differences in the chemical composition of the materials used, including protein and lipid fractions, which can ultimately induce changes in the type of interactions between the base flour and TF, WSF and DWSF supplements.

The quality and baking value of the flour, and thus its further use, can be described based on farinograph tests. According to a simple rating system proposed by Rohrlich and Brückner [55], wheat flour can be classified into “weak” (for confectionery) or “strong” (for bread making). In addition, “intermediate” flour with mixed characteristics is sometimes distinguished [53]. Based on the total rheological data obtained, the flour blends tested can therefore be described as either strong (such as a mixture of WF and DWSF material) or intermediate (such as a mixture of WF and 20−30% WSF material) (Table 6).

## 3. Materials and Methods

### 3.1. Chemicals and Reagents

*n*-Hexane, ethyl acetate, and methanol (all HPLC grade) were obtained from Fisher Chemical (Argenta, Poznań, Poland). A 2,2-diphenyl-1-picrylhydrazyl (DPPH) radical, 2,2′-azinobis-3-ethylbenzthiazoline-6-sulphonic acid (ABTS), ferrozine, Trolox, Folin-Ciocalteu reagent, gallic acid (≥98%), luteolin (>99%), EDTA, iron (II) chloride, potassium persulfate, sodium carbonate (all ACS grade), and acetonitrile and formic acid (both MS grade) were obtained from Merck (Merck, Warsaw, Poland). Melatonin and melatonin-*d*_4_ standards were purchased from Cayman Chemical (Biokom, Janki, Poland). Sulfuric, perchloric, and nitric acids, and hydrogen peroxide (all ACS grade) were purchased from Avantor (Gliwice, Poland). Ultrapure water was prepared with a Milli-Q purification system (Millipore, Burlington, MA, USA).

### 3.2. Plant Material

The research material consisted of randomly selected samples of whole white teff (https://www.teff-shop.de, Germany; accessed on 16 February 2020) and watermelon seeds (country of origin Hungary), and watermelon seed pomace flour (OlVita, Mysłaków, Poland) and wheat flour type 650 (Grain and Milling Company in Stoisław, Poland) purchased on the Polish market in 2020. Fresh watermelon seeds were collected from several ripe fruits and dried to a constant mass in a convection chamber drying oven (SML dryer, Zalmed, Łomianki, Poland) at 100 °C (seed water content was 47.4%). To obtain whole-grain flour, teff and watermelon seed samples were finely ground using a Lab Mill 120 laboratory mill (sieve of 800 µm was used; Perten Instruments, Germany). All samples were stored in a dry and shaded room until tests were conducted. The moisture content of test flours was estimated by lyophilization (Gamma 2–16 LSC, Christ, Osterode am Harz, Germany) of their representative samples: teff flour (TF, 9.68%), watermelon seed flour (WSF, 2.03%), and watermelon seed pomace flour (DWSF, 6.27%). Demonstrative photographs of raw and processed plant materials are shown in Figure S1.

### 3.3. Mineral Content

The material was mineralized with a mixture of H_2_SO_4_ and HClO_4_ or HNO_3_ and HClO_4_ for macro- and microelement determination, respectively. The total nitrogen was assessed based on a Kjeldahl method using Vapodest 30 apparatus (Gerhardt, Germany). Phosphorus was analyzed by the colorimetric method using a Specol 221 apparatus (866287, Carl Zeiss Jena, Germany) (ISO 6491:2000P) [56], while potassium, sodium, and calcium by emulsion flame spectroscopy, and magnesium, iron, zinc, manganese, lead, molybdenum, copper, and cadmium by absorption flame spectroscopy (spectrometer iCE 3000 Series, Thermo Fisher Scientific, Waltham, MA, USA) (ISO 6869:2000) [57].

### 3.4. Protein and Lipid Content

Crude protein was calculated from the total nitrogen using a conversion factor (N × 5.7) (AOAC 920.87-1920) [58]. Total lipid content was determined based on the *n*-hexane fraction obtained after extracting material in a Soxhlet apparatus.

### 3.5. Phytochemical Analyses

#### 3.5.1. Preparation of Extracts

The plant material was initially defatted with *n*-hexane under reflux (Soxhlet apparatus, 180 min) and then dried at room temperature for 12 h. Subsequently, one gram of defatted material was extracted twice with 80% MeOH (2 × 10 mL) using an ultrasonic bath (three cycles of 15 min each, 35 °C; Sonic-33, Polsonic, Poland). Extracts were evaporated to dryness at 40 °C under a vacuum, then reconstituted in 2 mL of 80% MeOH and stored at −20 °C until further use. The yield of extraction (% of material weight) was as follows: TF (4.1 ± 0.7%), WSF (13.2 ± 0.6%), and DWSF (6.0 ± 0.2%).

#### 3.5.2. Phytochemical Profiling and Quantification of Flavonoids Using UHPLC-PDA-MS/MS Analysis

Flour extracts were analyzed with an ACQUITY UPLC system equipped with a photodiode array detector (PDA) and a tandem quadrupole mass spectrometer (TQD-MS, Waters, Milford, MA, USA) using an electrospray ionization (ESI) source. The samples were chromatographed on the HSS C18 column (2.1 × 100 mm, 1.8 μm, Waters) maintained at 40 °C. Separations were carried out using a 12 min linear gradient (2→40%) of the acetonitrile–water mixture (both acidified with 0.1% formic acid), with a flow rate of 0.4 mL/min. The UV spectra were recorded within the range of 190–490 nm. The MS and MS/MS analyses were performed in both ionization modes, using the following settings: scan range 50–1200 *m*/*z*; capillary and cone voltages 2.8 and 3.1 kV (for ESI neg and ESI pos), and 45 and 60 V (for ESI neg and ESI pos); source and desolvation temperatures 150 and 450 °C; desolvation and cone gas flows 900 and 100 L/h; collision gas (argon) flow 0.1 mL/min; collision energy (CE) 28 V. Data acquisition and processing were performed using Waters MassLynx 4.1 software. Identification of chromatographic peaks was based on obtained LC-PDA-MS/MS data and subsequent comparison with the in-house metabolite database and the literature.

Flavonoids were determined using the above UPLC method with UV_345nm_ detection. Luteolin, the main flavonoid type in all materials, was selected as group standard. The luteolin calibration curve was in the range of 0.5–150 µg/mL and showed good linearity (R^2^ ≥ 0.999). Quantitative results were expressed as milligrams of standard equivalents (eq) per kilogram of plant material.

#### 3.5.3. Total Phenolic Content (TPC)

The total phenolic content was determined with the Folin–Ciocâlteu assay. Briefly, 0.1 mL of F-C reagent was added to 1.6 mL of an appropriately diluted sample (2.5–5.0 mg/mL) or gallic acid (GA) standard solution (1–10 µg/mL), then 0.3 mL of Na_2_CO_3_ (20% *w*/*v*) was added, and the mixture was incubated in 40 °C for 30 min. The Abs_765nm_ was measured using an Evolution 260 Bio spectrophotometer (Thermo Fisher, Waltham, MA, USA). The TPC was read from the linear curve of GA (R^2^ ≥ 0.99) and expressed as milligrams of GA eq/g of plant material.

#### 3.5.4. GC-MS Analysis

GC-MS analyses were performed with a non-defatted material and using an Agilent 6890N gas chromatograph combined with a mass selective detector (5973N MSD, Agilent Technologies, Santa Clara, CA, USA). Methanolic extracts of flours were prepared by sonication repeated twice (2 g × 5 mL × 60 min), and then chromatographed on an HP-5MSI column (30 m × 0.25 mm × 0.25 μm, Agilent). A temperature gradient program of 80→320 °C (temp. ramping rate—5 °C/min) was used along with a carrier gas (helium) flow of 1.2 mL/min. The sample injection volume was 2 μL. Mass spectra were recorded in the 20–600 *m*/*z* range using an electron impact ionization (70 eV) mode. Identification of chromatographic peaks was based on a comparison of GC-MS data with the NIST02 library and in-house metabolite database.

#### 3.5.5. Quantitative UHPLC-MS/MS Determination of Melatonin (MEL)

Test flours were extracted with pressurized solvent extractor ASE 200 (Dionex, Sunnyvale, CA, USA) using a method proposed by Setyaningsih et al. [59]. Briefly, 1 g of defatted material was mixed with 400 mg of diatomaceous earth and extracted twice with 70% EtOAc in MeOH at 100 °C and 1500 psi. A 40 ng of internal standard (IS, MEL-*d*_4_) was added to each sample before extraction. Extracts were evaporated at 40 °C and reconstituted in 2 mL of 90% MeOH. A seven-point calibration curve (1–50 ng/mL) of MEL was prepared with 90% MeOH, and the content of IS was fixed to 20 ng/mL.

Quantitative analyses were performed using the UPLC system coupled to the ESI-TQD-MS (Waters). The samples were chromatographed on the HSS C18 column (2.1 × 100 mm, 1.8 μm, Waters) maintained at 40 °C. Separations were carried out using a 6.5 min linear gradient (5→30%) of the acetonitrile–water mixture (both acidified with 0.1% formic acid), with a flow rate of 0.4 mL/min. The injection volume was 2 μL. The MS analyses were performed in positive ion mode, and the following instrumental parameters were used: capillary and cone voltages—3.0 kV and 30 V; source and desolvation temperatures—150 and 450 °C, desolvation and cone gas flow—900 and 100 L/h. The detection of MEL and MEL-d4 was performed in selected reaction monitoring (SRM) mode using the following transitions: 233→159 (CE 30 V), 233→174 (CE 10 V), and 237→163 (CE 30 V), 237→178 (CE 10 V), respectively [60]. Waters MassLynx v.4.1 software was used for data acquisition and processing. Results are expressed as micrograms per kilogram of plant material.

### 3.6. Antioxidant Properties of Teff and Watermelon Material

Antioxidant tests were performed with the use of aqueous-methanolic extracts prepared according to Section 3.5.1.

#### 3.6.1. ABTS Assay

The ABTS test was carried out according to Kontek et al. [61]. The radical cations (ABTS+) were prepared by mixing an equal volume of 7 mM ABTS and 4.9 mM potassium persulfate. The working solution (Abs_734nm_ = 0.7 AU) was obtained by dilution with 50% MeOH. Concentrations of test extracts of TF (50–200 mg of material/mL), WSF (30–120 mg/mL) and DWSF (20–80 mg/mL), and Trolox (10−250 μg/mL) were prepared with 50% MeOH. A 30 μL of extract/standard was mixed with 1.5 mL of ABTS+ solution, and after 30 min, the Abs_734nm_ was measured using a UV-vis spectrophotometer (Evolution 260 Bio, Thermo Fisher). The absorbance inhibition (%) was calculated as follows: [(Abs_control_–Abs_sample_)/Abs_control_] × 100. Results were determined from the linear curves (absorbance inhibition (%) vs. concentration (µg/mL)) of samples and are expressed as IC_50_ values, defined as the concentration necessary to cause 50% radical inhibition.

#### 3.6.2. DPPH Assay

The DPPH antiradical test was carried out according to Kontek et al. [61]. A 1.9 mL quantity of DPPH methanolic solution (100 µM) was mixed with 0.1 mL of the extract (20–150 mg of material/mL) or Trolox solution (10–200 µg/mL). After 30 min incubation, the Abs_517nm_ was measured using the Evolution 260 Bio spectrophotometer. Results were calculated and expressed as reported in Section 3.6.1.

#### 3.6.3. Ferrous Ion Chelating Activity (FCA)

The FCA test was carried out according to Rahman et al. [62]. Briefly, a 250 µL of extract/positive control (EDTA), 1.6 mL of PBS (0.75 M, pH 7.0), 25 µL of FeCl_2_ solution (2 mM), and 100 µL of ferrozine solution (5 mM) were added to the test tube, mixing well each time, and after 15 min the Abs_562nm_ was measured using the Evolution 260 Bio spectrophotometer. Four concentrations of each extract—TF (50–250 mg/mL), WSF (25–150 mg/mL), and DWSF (50–200 mg/mL)—and five concentrations of EDTA (10−130 μg/mL) were tested. Results were calculated and expressed as reported in Section 3.6.1.

### 3.7. Farinographic Evaluation of Dough Based on Wheat Flour with Test Supplements

The rheological properties of dough, including water absorption (%), development time (min), stability time (min), softening (ICC-12 min after peak time and 10 min after beginning, FU), and farinograph quality number (mm), were determined using a farinograph rheometer (Farinograph-E with USB port, Brabender, Germany) according to PN-EN ISO 5530-1:2015-01E method [63]. Teff and watermelon seed flours were mixed with wheat flour type 650 in the proportions of 10%, 20%, and 30% (*w*/*w*); higher levels of test samples harmed the sensory properties of the final product (preliminary results). The size of the flour blend was 50 g (14% moisture basis).

### 3.8. Statistical Analyses

Experiments were performed in triplicate at minimum, and results are expressed as means ± standard deviations (SD). Statistical comparison of data was performed using one-way ANOVA, followed by Tukey’s comparison test. Significance was considered at *p* < 0.05. Data were analyzed using Statistica 13.0 (Statsoft Inc, Tulsa, OK, USA) and Microsoft Excel software.

## 4. Conclusions

In this work, applicability and farinographic evaluation of teff (TF) and two watermelon seed flours (pomace (DWSF) and seeds (WSF)) as baking supplements was carried out. Farinographic studies were performed using supplementation of refined flour with 10–30% levels of additives and measuring important rheological parameters of the dough. The most affected farinographic traits were development time, quality number, and softening. Overall, the best results were achieved after supplementation with watermelon seed pomace. In addition, the DWSF material had the highest levels of P, Mg, Na, Fe, Zn, and Mo. Protein and fat levels in watermelon seed materials were two- to tenfold those in TF. Phytochemical profiling highlighted the abundance of phenolic compounds in test flours, including flavone glycosides in teff, and flavone aglycones in watermelon seeds. However, total polyphenol values were low in all materials (<2 mg GAE/g), which also correlates with the low antioxidant potential of the samples oforbtained in ABTS and DPPH assays. Watermelon seed samples, especially pomace, were characterized by significantly higher melatonin concentration (DWSF, 65 µg/kg) than TF (3.5 µg/kg). Obtained results provide new information about the chemical composition of teff and watermelon seeds and prove that these materials can be a valuable supplement to refined flours, improving their quality and baking value.

## Figures and Tables

**Table 1:** Mineral, protein, and fat content in teff and watermelon seed flour samples (means ± SD).

	Parameter	Teff	Watermelon Seeds	Watermelon Seed Pomace
Macroelements	P (g·kg^−1^ dm ^a^)	5.40 ± 0.1 c	6.05 ± 0.1 b	7.50 ± 0.1 a
	K (g·kg^−1^ dm)	2.71 ± 0.1 c	6.31 ± 0.0 a	5.14 ± 0.1 b
	Ca (g·kg^−1^ dm)	0.89 ± 0.0 a	0.35 ± 0.0 c	0.50 ± 0.0 b
	Mg (g·kg^−1^ dm)	1.53 ± 0.0 b	1.54 ± 0.0 b	1.72 ± 0.0 a
	Na (g·kg^−1^ dm)	0.08 ± 0.0 b	0.10 ± 0.0 b	0.44 ± 0.0 a
Microelements	Fe (mg·kg^−1^ dm)	60.30 ± 0.7 b	28.70 ± 0.9 c	124.20 ± 0.2 a
	Zn (mg·kg^−1^ dm)	22.90 ± 0.1 c	25.20 ± 0.0 b	27.50 ± 0.0 a
	Mn (mg·kg^−1^ dm)	42.60 ± 0.4 a	16.10 ± 0.2 b	13.80 ± 0.3 c
	Cu (mg·kg^−1^ dm)	nd ^b^	nd	nd
	Mo (mg·kg^−1^ dm)	13.80 ± 0.7 b	12.50 ± 0.7 b	17.50 ± 0.3 a
Toxic metals	Pb (mg·kg^−1^ dm)	nd	nd	nd
	Cd (mg·kg^−1^ dm)	nd	nd	nd
	Crude protein (%)	11.70 ± 0.1 c	20.50 ± 0.1 b	25.20 ± 0.1 a
	Total lipids (%)	2.87 ± 0.0 c	29.61 ± 0.5 a	8.99 ± 0.1 b

^a^ dm—dry mass; ^b^ nd—not detected. Mean values with the same letter in each row are not significantly different (Tukey’s test, *p* < 0.05). Color code was generated using Microsoft Excel and indicates mineral levels in the test flours (green (high)→white (medium)→red (low)).

**Table 2:** Phytochemical screening of teff and watermelon flour samples based on UHPLC-PDA-ESI-MS/MS analysis.

No	RT (min)	UVmax (nm)	[M-H]^−^, *m*/*z*	MS/MS Fragments ^a^	[M+H] ^+^, *m*/*z*	MS/MS Fragments ^a^	Identity	Presence in Sample ^b^		
								Teff Flour	Watermelon Seed Flour	Watermelon Seed Pomace Flour
1	5.12	255, 345	609	327, 357, 411	611	329, 431, 449	luteolin di-hex ^d^	+	nd	nd
2	5.20	287, 325	355	175, 160, 193	379 ^c^/357	-	ferulic acid hex	nd	+	+
3	5.22	255, 269, 348	609	327, 357, 411	611	329, 299, 353	luteolin di-hex	+++	nd	nd
4	5.54	295sh, 325	401	101, 71, 161	425 ^c^	-	unidentified	nd	trace	+
5	5.78	269, 335	593	311, 341	595	313, 283, 397	apigenin di-hex	+	nd	nd
6	6.00	269, 348	447	357, 327, 297	449	299, 329	luteolin *C*-hex	+	trace	+
7	6.06	271, 335	623	341, 371	625	343, 367, 313	methoxyluteolin di-hex	+	nd	nd
8	6.19	256, 269, 350	447	327, 357, 297	449	329, 299, 413	luteolin *C*-hex	+++	+	+
9	6.70	268, 336	431	311, 283, 341	433	313, 283, 397	apigenin *C*-hex	+	++	+++
10	6.80	255, 269, 348	593	285	595	287, 449	luteolin *O*-deoxyhex-hex	+	nd	nd
11	6.99	255, 269, 348	447	447, 285	449	287	luteolin *O*-hex	++	nd	nd
12	7.11	264, 347	759	327, 357, 411	761	151, 329, 431	luteolin *O*-syringyl-pentoside *C*-hex	+++	nd	nd
13	7.67	339	177	162	179	91, 79, 146	hydroxy-methoxycinnamyl aldehyde	nd	++	+
14	7.75	265, 341	447	285	449	287	luteolin-7-*O*-glucoside ^#^	nd	+++	++
15	7.82	269, 348	489	327, 299, 357	491	329, 299, 311	luteolin *C*-acetylhex	+	nd	nd
16	7.90	339	177	162, 134	179	91, 79, 119	hydroxy-methoxycinnamyl aldehyde	nd	+	+
17	8.01	255, 345	461	283, 446, 298	463	301	methoxyluteolin *O*-hex	trace	trace	+
18	8.91	277	339	263, 291, 327	341	137, 251	unidentified	nd	+	trace
19	9.40	255, 269, 347	285	133, 151, 175	287	287, 153, 135	luteolin ^#^	nd	+++	+++
20	9.79	269, 345	551	473, 165, 503	575 ^c^/553	-	unidentified bi-flavonoid	nd	+	trace
21	9.92	269, 345	551	165, 325, 195	575 ^c^/553	-	unidentified bi-flavonoid	nd	+	trace
22	10.11	345	337	279, 307, 291	339	137, 219, 161	unidentified	nd	+++	++
23	10.55	267, 337	269	117, 151, 149	271	271, 153, 119	apigenin ^#^	nd	+	+++
24	10.88	255, 269, 350	299	284, 256	301	286, 258, 301	chrysoeriol ^#^	nd	++	+++
25	11.11	315	383	163, 119, 145	407 ^c^/385	147	di-coumaroyl-glycerol	+	nd	nd
26	11.37	320	413	163, 193, 145	437 ^c^	147	coumaroyl-feruloyl-glycerol	+	nd	nd
27	11.49	220, 345	515	219, 467, 485	539 ^c^	137, 427, 455	unidentified	nd	+	trace

^a^ Base peak ion underlined; ^b^ presence in the sample (based on peak area at the UV_330nm_ chromatogram): nd—not detected, trace—trace signal, +—minor component, ++—intermediate component, +++—major component; ^c^ [M+Na]^+^ ion; ^d^ hex—hexoside; ^#^ identity confirmed based on comparison with authentic standard compound.

**Table 3:** Flavonoid, total phenolic (TPC), and melatonin content in teff and watermelon flour material (mean ± SD).

No	Compound	Content (mg Luteolin eq/kg of Plant Material)		
		Teff Flour (TF)	Watermelon Seed Flour (WSF)	Watermelon Seed Pomace Flour (DWSF)
1	luteolin di-hex	3.03 ± 0.17	nd	nd
2	luteolin di-hex	70.86 ± 1.14	nd	nd
3	apigenin di-hex	8.12 ± 0.44	nd	nd
4	luteolin *C*-hex	6.66 ± 0.61 a	trace	5.92 ± 0.37
5	methoxyluteolin di-hex	5.35 ± 0.70	nd	nd
6	luteolin *C*-hex	91.41 ± 1.01 c	1.71 ± 0.29 a	6.01 ± 0.30 b
7	apigenin *C*-hex	7.53 ± 0.27 a	18.63 ± 1.23 b	102.94 ± 6.20 c
8	luteolin *O*-deoxyhex-hex	11.16 ± 0.27	nd	nd
9	luteolin *O*-hex	10.90 ± 0.39	nd	nd
10	luteolin *O*-syringyl-pentoside *C*-hex	28.53 ± 0.73	nd	nd
11	luteolin-7-*O*-glucoside	nd	10.65 ± 0.57 a	37.03 ± 2.14 b
12	luteolin *C*-acetylhex	4.87 ± 0.16	nd	nd
13	methoxyluteolin *O*-hex	trace	trace	4.79 ± 0.50
14	luteolin	nd	42.51 ± 1.19 a	372.07 ± 20.64 b
15	unidentified flavonoid	nd	1.57 ± 0.21	trace
16	unidentified flavonoid	nd	2.27 ± 0.29	trace
17	apigenin	nd	6.07 ± 0.54 a	91.17 ± 4.89 b
18	chrysoeriol	nd	9.46 ± 0.45 a	101.14 ± 5.89 b
	total flavonoids (mg luteolin eq/kg)	243.56 ± 3.27 b	92.87 ± 3.66 a	721.09 ± 39.64 c
	total phenolic content (mg GAE/g)	0.51 ± 0.03 a	1.01 ± 0.03 b	1.67 ± 0.03 c
	melatonin (µg/kg)	3.50 ± 0.24 a	11.29 ± 0.48 b	63.33 ± 1.28 c

Mean values with the same letter in each row are not significantly different (Tukey’s test, *p* < 0.05).

**Table 4:** Compounds identified in methanolic extracts of teff and watermelon flour samples by GC-MS analysis.

No	Compound	RT (min)	MS Signals *, *m*/*z*		Presence in Sample **		
			[M^●^] ^+^	Characteristic Fragment Ions	Teff Flour	Watermelon Seed Flour	Watermelon Seed Pomace Flour
1	unidentified	13.31	164(?)	29, 31, 57, 73, 43	nd	nd	++
2	unidentified	14.13	164(?)	57, 29, 31, 73, 42	+++	nd	nd
3	Palmitic acid	23.54	256	43, 73, 60, 41, 57	nd	+	nd
4	Linoleic acid	27.51	280	67, 81, 82, 95, 55	nd	+++	nd
5	(*Z*)-9-Octadecenamide	30.33	281	59, 72, 55, 41, 43	trace	trace	+
6	unidentified	35.08	410(?)	117, 131, 67, 41, 81	trace	nd	++
7	unidentified	35.40	410(?)	67, 55, 81, 117, 95	nd	++	nd
8	Squalene	37.00	410	69, 81, 41, 136, 137	nd	+	+
9	β-Tocopherol	40.06	416	151, 43, 191, 55, 57	+	+	+
10	Stigmasterol	42.65	412	55, 43, 81, 69, 83	nd	+	+
11	β-Sitosterol	43.33	414	43, 55, 41, 57, 107	++	nd	nd
12	Isomultiflorenon	43.74	424	205, 257, 245, 121, 119	nd	+	+
13	β-Amyrin	44.22	426	218, 203, 219, 189, 95	+	nd	nd
14	Lupeol	44.23	426	68, 55, 67, 81, 95	nd	trace	trace

* Base peak ion underlined; (?) supposed molecular ion; ** presence in the sample (based on peak area in total ion chromatogram): nd—not detected, trace—trace signal, +—minor component, ++—intermediate component, +++—major component.

**Table 5:** Antiradical and metal chelating activity in vitro of aqueous methanolic extracts of teff and watermelon material using 2,2′-azinobis-3-ethylbenzthiazoline-6-sulphonic acid (ABTS) and 2,2-diphenyl-1-picrylhydrazyl (DPPH) radical and ferrous ion chelating (FCA) assays (mean ± SD).

Sample	IC_50_ (mg of Plant Material/mL)		
	ABTS Assay	DPPH Assay	FCA Assay
Teff flour (TF)	156.72 ± 0.47 d (6.62 ± 0.02) #	73.93 ± 0.48 c (3.04 ± 0.03)	155.58 ± 1.70 d (5.16 ± 0.03)
Watermelon seed flour (WSF)	116.66 ± 0.61 c (15.73 ± 0.38)	107.68 ± 1.49 d (15.38 ± 0.21)	95.25 ± 4.47 b (11.55 ± 0.54)
Watermelon seed pomace flour (DWSF)	78.60 ± 1.18 b (4.52 ± 0.08)	45.97 ± 0.63 b (2.68 ± 0.04)	114.43 ± 7.16 c (6.35 ± 0.16)
Control positive (Trolox * or EDTA **)	0.14 ± 0.00 *a	0.10 ± 0.00 *a	0.06 ± 0.00 **a

Mean values with the same letter in a column are not significantly different (Tukey’s test, *p* < 0.05). # Values in parentheses correspond to the IC_50_ activity of the sample expressed in mg extract/mL. * Trolox is the positive control; ** EDTA is the positive control.

**Table 6:** Rheological properties of dough based on wheat flour with the addition of teff and watermelon seed flours (test levels: 10%, 20% and 30% *w*/*w*). (mean ± SD).

Sample	Farinograph Parameter					Flour Quality
	Water Absorption (%)	Development Time (min)	Stability Time (min)	Degree of Softening (after 10 min, FU)	Farinograph Quality Number (mm)	
WF ^a^ + 10% TF ^b^	57.3 ± 0.2 bc	4.6 ± 0.1 ab	7.5 ± 0.1 bc	37.0 ± 0.0 e	88 ± 0.1 b	strong
WF + 20% TF	56.9 ± 0.2 c	4.5 ± 0.1 ab	5.9 ± 0.0 d	50.0 ± 0.0 b	81 ± 0.7 c	strong
WF + 30% TF	56.6 ± 0.2 cd	3.9 ± 0.5 bc	4.2 ± 0.1 e	71.5 ± 0.7 a	60 ± 0.0 e	medium
WF + 10% WSF ^c^	56.8 ± 0.2 cd	5.5 ± 0.5 ab	8.5 ± 0.1 ab	28.5 ± 0.7 f	82 ± 0.0 c	strong
WF + 20% WSF	56.1 ± 0.0 de	4.3 ± 0.1 abc	6.1 ± 0.0 d	53.5 ± 0.7 b	73 ± 0.7 d	medium
WF + 30% WSF	56.6 ± 0.2 cd	3.9 ± 0.6 bc	4.3 ± 0.1 e	70.0 ± 0.7 a	63 ± 0.1 e	medium
WF + 10% DWSF ^d^	57.2 ± 0.0 bc	6.0 ± 0.7 a	7.2 ± 0.1 c	37.5 ± 0.7 e	77 ± 0.0 d	strong
WF + 20% DWSF	57.9 ± 0.4 ab	5.5 ± 0.7 ab	6.9 ± 0.1 cd	40.5 ± 0.7 d	78 ± 0.1 cd	strong
WF + 30% DWSF	58.7 ± 0.0 a	4.9 ± 0.7 ab	8.9 ± 0.7 a	44.0 ± 0.7 c	100 ± 0.0 a	strong
WF (control)	55.4 ± 0.3 e	2.6 ± 0.1 c	6.8 ± 0.2 cd	51.5 ± 0.7 b	62 ± 0.0 e	medium

Samples: ^a^ WF—wheat flour; ^b^ TF—teff flour; ^c^ WSF—watermelon seed flour; ^d^ DWSF—watermelon seed pomace flour. Mean values with the same letter in each column are not significantly different (Tukey’s test, *p* < 0.05).

## Data Availability

Data associated with this work will be freely available upon request.

## Supplementary Materials

The following supporting information can be downloaded at https://www.mdpi.com/article/10.3390/molecules28073255/s1. Figure S1: Demonstrative photographs of raw and processed test materials: teff seeds (A), teff flour (B), watermelon seeds (C), watermelon whole seed flour prepared in-house (D), and commercial watermelon seed pomace (defatted) flour (E) (photographs were taken by D. Jedrejek and M. Sobolewska); Figure S2: Effect of the addition of teff and watermelon seed flours (test levels: 10%, 20% and 30% *w*/*w*) to wheat flour on the rheological properties of the dough. All results were normalized to a control sample (wheat flour type 650) = 1.0. Samples: WF—wheat flour, TF—teff flour, WSF—watermelon seed flour, DWSF—watermelon seed pomace flour; Figure S3: UHPLC-UV-MS chromatograms of methanolic extract of teff flour; Figure S4: UHPLC-UV-MS chromatograms of methanolic extract of watermelon seed flour; Figure S5: UHPLC-UV-MS chromatograms of methanolic extract of watermelon seed pomace flour; Figure S6: GC-MS chromatogram of methanolic extract of teff flour; Figure S7: GC-MS chromatogram of methanolic extract of watermelon seed flour; Figure S8: GC-MS chromatogram of methanolic extract of watermelon seed pomace flour; Figure S9: LC-MS/MS chromatograms of melatonin (MEL) and melatonin *d*_4_ (MEL-*d*_4_) standards—(A)), and blank sample—(B)); Figure S10: LC-MS/MS chromatograms of MEL and MEL-*d*_4_ from test samples: teff flour—(A)), watermelon seed flour—(B)), watermelon seed pomace flour—(C)); Figure S11: Inhibition curves (inhibition% vs sample concentration) obtained for test samples and positive controls (Trolox or EDTA) in antiradical (ABTS and DPPH) and metal ion chelating (FCA) assays; Table S1: Current literature data on the content of minerals as well as total protein and fat in teff and watermelon seeds and/or flours; Table S2: Correlations between the analyzed rheological parameters; Table S3: Current literature data on the total phenolic content and antioxidant activity of teff and watermelon seeds and/or flours; Table S4: Calibration parameters and results of the evaluation of linearity (regression coefficient (R2), goodness-of-fit (gof), means ± standard deviation), limit of detection (LOD), limit of quantification (LOQ), reproducibility (relative standard deviation—RSD), and recovery (means ± standard deviation) of melatonin determination in teff, watermelon seed flour and watermelon seed pomace flour. References [64,65,66,67,68,69,70,71,72,73] are cited in the Supplementary Materials (Tables S1 and S3).

## References

[1] Ertl K., Goessler W. (2018). Grains, whole flour, white flour, and some final goods: An elemental comparison. Eur. Food Res. Technol..

[2] Wang Y., Jian C. (2022). Sustainable plant-based ingredients as wheat flour substitutes in bread making. Npj Sci. Food.

[3] Slavin J.L., Jacobs D., Marquart L. (2001). Grain Processing and Nutrition. Crit. Rev. Biotechnol..

[4] Oghbaei M., Prakash J. (2016). Effect of primary processing of cereals and legumes on its nutritional quality: A comprehensive review. Cogent Food Agric..

[5] Saleh A.S., Wang P., Wang N., Yang S., Xiao Z. (2019). Technologies for enhancement of bioactive components and potential health benefits of cereal and cereal-based foods: Research advances and application challenges. Crit. Rev. Food Sci. Nutr..

[6] Wang J., Chatzidimitriou E., Wood L., Hasanalieva G., Markellou E., Iversen P.O., Seal C., Baranski M., Vigar V., Ernst L. (2020). Effect of wheat species (*Triticum aestivum* vs. *T. spelta*), farming system (organic vs conventional) and flour type (wholegrain vs white) on composition of wheat flour—Results of a retail survey in the UK and Germany—2. Antioxidant activity, and phenolic and mineral content. Food Chem..

[7] Zuchowski J., Jonczyk K., Pecio L., Oleszek W. (2011). Phenolic acid concentrations in organically and conventionally cultivated spring and winter wheat. J. Sci. Food Agric..

[8] Mancebo C.M., Picón J., Gómez M. (2015). Effect of flour properties on the quality characteristics of gluten free sugar-snap cookies. LWT Food Sci. Technol..

[9] Conte P., Fadda C., Drabińska N., Krupa-Kozak U. (2019). Technological and Nutritional Challenges, and Novelty in Gluten-Free Breadmaking—A Review. Pol. J. Food Nutr. Sci..

[10] Dziki D., Różyło R., Gawlik-Dziki U., Świeca M. (2014). Current trends in the enhancement of antioxidant activity of wheat bread by the addition of plant materials rich in phenolic compounds. Trends Food Sci. Technol..

[11] Garutti M., Nevola G., Mazzeo R., Cucciniello L., Totaro F., Bertuzzi C.A., Caccialanza R., Pedrazzoli P., Puglisi F. (2022). The Impact of Cereal Grain Composition on the Health and Disease Outcomes. Front. Nutr..

[12] Zięć G., Gambuś H., Lukasiewicz M., Gambuś F. (2021). Wheat Bread Fortification: The Supplement of Teff Flour and Chia Seeds. Appl. Sci..

[13] Saeid A., Ahmed M., Novo de Barros A., Gouvinhas I. (2021). A Review on Effects of Pseudo Cereals Flour on Quality Properties of Biscuit, Cookies and Cake. Innovation in the Food Sector Through the Valorization of Food and Agro-Food By-Products.

[14] Katyal M., Kaur A., Singh N. (2019). Effects of incorporation of groundnut oil and hydrogenated fat on pasting and dough rheological properties of flours from wheat varieties. J. Food Sci. Technol..

[15] Mihiretu A., Asresu M. (2022). Inclusive technology performance evaluation in the production of teff (*Eragrostis tef* (Zucc.) Trotter). Adv. Agric..

[16] Bultosa G., Taylor J.R.N., Wrigley C., Corke H., Walker C. (2004). Teff. Encyclopedia of Grain Science.

[17] Hager A.S., Wolter A., Jacob F., Zannini E., Arendt E.K. (2012). Nutritional properties and ultra-structure of commercial gluten free flours from different botanical sources compared to wheat flours. J. Cereal Sci..

[18] World Watermelon Production by Country. https://www.atlasbig.com/en-us/countries-watermelon-production.

[19] El-Adawy T.A., Taha K.M. (2001). Characteristics and Composition of Watermelon, Pumpkin, and Paprika Seed Oils and Flours. J. Agric. Food Chem..

[20] Mahla H.R., Rathore S.S., Venkatesan K., Sharma R. (2018). Analysis of fatty acid methyl esters and oxidative stability of seed purpose watermelon (*Citrullus lanatus*) genotypes for edible oil. J. Food Sci. Technol..

[21] Dias M.C., Pinto D.C.G.A., Silva A.M.S. (2021). Plant Flavonoids: Chemical Characteristics and Biological Activity. Molecules.

[22] Ou J., Wang M., Zheng J., Ou S. (2019). Positive and negative effects of polyphenol incorporation in baked foods. Food Chem..

[23] Manivannan A., Lee E.-S., Han K., Lee H.-E., Kim D.-S. (2020). Versatile Nutraceutical Potentials of Watermelon—A Modest Fruit Loaded with Pharmaceutically Valuable Phytochemicals. Molecules.

[24] Conforti F.D., Johnson J.M. (1992). Use of the Farinograph in Predicting Baking Quality of Unchlorinated and Chlorinated Flours. J. Food Qual..

[25] Katyal M., Kaur A., Singh N. (2019). Evaluation of pasting and dough rheological properties of composite flours made from flour varied in gluten strength. J. Food Sci. Technol..

[26] Coleman J., Abaye A.O., Barbeau W., Thomason W. (2013). The suitability of teff flour in bread, layer cakes, cookies and biscuits. Int. J. Food Sci. Nutr..

[27] Anang D.A., Pobee R.A., Antwi E., Obeng E.M., Atter A., Ayittey F.K., Boateng J.T. (2018). Nutritional, microbial and sensory attributes of bread fortified with defatted watermelon seed flour. Int. J. Food Sci..

[28] Wani A.A., Sogi D.S., Singh P., Khatkar B.S. (2015). Influence of watermelon seed protein concentrates on dough handling, textural and sensory properties of cookies. J. Food Sci. Technol..

[29] USDA FoodData Central. https://fdc.nal.usda.gov/fdc-app.html#/food-details/169407/nutrients.

[30] Salawu S.O., Bester M.J., Duodu K.G. (2013). Phenolic composition and bioactive properties of cell wall preparations and whole grains of selected cereals and legumes. J. Food Biochem..

[31] Shumoy H., Raes K. (2016). Antioxidant Potentials and Phenolic Composition of Tef Varieties: An Indigenous Ethiopian Cereal. Cereal Chem..

[32] Kotaskova E., Sumczynski D., Mlcek J., Valasek P. (2016). Determination of free and bound phenolics using HPLC-DAD, antioxidant activity and *in vitro* digestibility of *Eragrostis tef*. J. Food Composit. Anal..

[33] Ravisankar S., Abegaz K., Awika J.M. (2018). Structural profile of soluble and bound phenolic compounds in teff (*Eragrostis tef*) reveals abundance of distinctly different flavones in white and brown varieties. Food Chem..

[34] Abu-Reidah I.M., Arráez-Román D., Segura-Carretero A., Fernández-Gutiérrez A. (2013). Profiling of phenolic and other polar constituents from hydro-methanolic extract of watermelon (*Citrullus lanatus*) by means of accurate-mass spectrometry (HPLC–ESI–QTOF–MS). Food Res. Int..

[35] Sorokina M., McCaffrey K.S., Deaton E.E., Ma G., Ordovas J.M., Perkins-Veazie P.M., Steinbeck C., Levi A., Parnell L.D. (2021). A Catalog of Natural Products Occurring in Watermelon—*Citrullus lanatus*. Front. Nutr..

[36] Tabiri B., Agbenorhevi J.K., Wireko-Manu F.D., Ompouma E.I. (2016). Watermelon Seeds as Food: Nutrient Composition, Phytochemicals and Antioxidant Activity. Int. J. Nutr. Food Sci..

[37] Fadimu G.J., Ghafoor K., Babiker E.E., Al-Juhaimi F., Abdulraheem R.A., Adenekan M.K. (2020). Ultrasound-assisted process for optimal recovery of phenolic compounds from watermelon (*Citrullus lanatus*) seed and peel. J. Food Meas. Charact..

[38] Oghabei M., Prakash J. (2019). Bioaccessible phenolics and flavonoids from wheat flour products subjected to different processing variables. Cereal Chem..

[39] Lu Y., Luthria D., Fuerst E.P., Kiszonas A.M., Yu L., Morris C.F. (2014). Effect of Processing on Phenolic Composition of Dough and Bread Fractions Made from Refined and Whole Wheat Flour of Three Wheat Varieties. J. Agric. Food Chem..

[40] Gil J.V., Esteban-Munoz A., Fernandez-Espinar M.T. (2022). Changes in the Polyphenolic Profile and Antioxidant Activity of Wheat Bread after Incorporating Quinoa Flour. Antioxidants.

[41] Lis B., Jedrejek D., Rywaniak J., Soluch A., Stochmal A., Olas B. (2020). Flavonoid Preparations from *Taraxacum officinale* L. Fruits—A Phytochemical, Antioxidant and Hemostasis Studies. Molecules.

[42] El-Alfy T.S., Ezzat S.M., Sleem A.A. (2012). Chemical and biological study of the seeds of *Eragrostis tef* (Zucc.) Trotter. Nat. Prod. Res..

[43] Jäger S., Trojan H., Kopp T., Laszczyk M.N., Scheffler A. (2009). Pentacyclic Triterpene Distribution in Various Plants – Rich Sources for a New Group of Multi-Potent Plant Extracts. Molecules.

[44] Tan D.-X., Hardeland R., Manchester L.C., Korkmaz A., Ma S., Rosales-Corral S., Reiter R.J. (2012). Functional roles of melatonin in plants, and perspectives in nutritional and agricultural science. J. Exp. Bot..

[45] Meng X., Li Y., Li S., Zhou Y., Gan R.-Y., Xu D.-P., Li H.-B. (2017). Dietary sources and bioactivities of melatonin. Nutrients.

[46] Navara K.J., Nelson R.J. (2007). The dark side of light at night: Physiological, epidemiological, and ecological consequences. J. Pineal Res..

[47] Sae-Teaw M., Johns J., Johns N.P., Subongkot S. (2013). Serum melatonin levels and antioxidant capacities after consumption of pineapple, orange, or banana by healthy male volunteers. J. Pineal Res..

[48] Santos J.S., Brizola V.R., Granato D. (2017). High-throughput assay comparison and standardization for metal chelating capacity screening: A proposal and application. Food Chem..

[49] Jedrejek D., Pawelec S., Piwowarczyk R., Pecio Ł., Stochmal A. (2020). Identification and occurrence of phenylethanoid and iridoid glycosides in six Polish broomrapes (*Orobanche* spp. and *Phelipanche* spp., Orobanchaceae). Phytochemistry.

[50] Forsido S.F., Rupasinghe H.P.V., Astatkie T. (2013). Antioxidant capacity, total phenolics and nutritional content in selected ethiopian staple food ingredients. Int. J. Food Sci. Nutr..

[51] Miladinović B., Ilić K., Stojanović D., Kostić M., Milutinović M., Branković S., Kitić D. (2020). Antioxidant activity, total phenol and tannin content of different varieties of flours. Acta Med. Median..

[52] Sedej I., Sakać M., Mandić A., Misan A., Tumbas V., Hadnadev M. (2011). Assessment of antioxidant activity and rheological properties of wheat and buckwheat milling fractions. J. Cereal Sci..

[53] Biel W., Jaroszewska A., Stankowski S., Sobolewska M., Kępińska-Pacelik J. (2021). Comparison of yield, chemical composition and farinograph properties of common and ancient wheat grains. Eur. Food Res. Technol..

[54] Lacko-Bartošová M., Konvalina P., Lacko-Bartošová L., Štěrba Z. (2019). Quality evaluation of emmer wheat genotypes based on rheological and Mixolab parameters. Czech J. Food Sci..

[55] Rohrlich M., Bruckner G. (1966). Cereals.

[56] (1998). Animal Feeding Stuffs—Determination of Phosphorus Content—Spectrometric Method.

[57] (2000). Animal Feeding Stuffs—Determination of The Contents of Calcium, Copper, Iron, Magnesium, Manganese, Potassium, Sodium and Zinc—Method Using Atomic Absorption Spectrometry.

[58] (2001). Protein (Total) in Flour.

[59] Setyaningsih W., Saputro I.E., Barbero G.F., Palma M., Barroso C.G. (2015). Determination of melatonin in rice (*Oryza sativa*) grains by pressurized liquid extraction. J. Agric. Food Chem..

[60] Gomez-Gomez A., Montenero-San-Martin B., Haro N., Pozo O.J. (2021). Nail Melatonin Content: A Suitable Non-Invasive Marker of Melatonin Production. Int. J. Mol. Sci..

[61] Kontek B., Jedrejek D., Oleszek W., Olas B. (2021). Antiradical and antioxidant activity in vitro of hops-derived extracts rich in bitter acids and xanthohumol. Ind. Crops Prod..

[62] Rahman M.J., de Camargo A., Shahidi F. (2017). Phenolic and polyphenolic profiles of chia seeds and their in vitro biological activities. J. Func. Foods.

[63] (2015). Wheat Flour—Physical Characteristics of Doughs—Part 1: Determination of Water Absorption and Rheological Properties Using a Farinograph.

[64] Bultosa G. (2007). Physicochemical Characteristics of Grain and Flour in 13 Tef [*Eragrostis tef* (Zucc.) Trotter] Grain Varieties. Res. J. Appl. Sci..

[65] Baye K., Mouquet-Rivier C., Icard-Verniere C., Picq C., Guyot J.-P. (2014). Changes in mineral absorption inhibitors consequent to fermentation of Ethiopian *injera*: Implications for predicted iron bioavailability and bioaccessibility. Int. J. Food Sci..

[66] Alemneh S.T., Emire S.A., Hitzmann B., Zettel V. (2022). Comparative Study of Chemical Composition, Pasting, Thermal and Functional properties of Teff (*Eragrostis tef*) Flours Grown in Ethiopia and South Africa. Int. J. Food Prop..

[67] USDA FoodData Central. https://fdc.nal.usda.gov/fdc-app.html#/food-details/169747/nutrients.

[68] Rezig L., Chouaibi M., Meddeb W., Msaada K., Hamdi S. (2019). Chemical composition and bioactive compounds of Cucurbitaceae seeds: Potential sources for new trends of plant oils. Process Saf. Environ. Prot..

[69] Kausar T., Hassan M.T., Din G.M. (2020). Utilization of watermelon seed flour as protein supplement in cookies. Pure Appl. Biol..

[70] Eke R., Ejiofor E., Oyedemi S., Onoja S., Omeh N. (2021). Evaluation of nutritional composition of *Citrullus lanatus* Linn. (watermelon) seed and biochemical assessment of the seed oil in rats. Food Biochem..

[71] Japu J.A., Ahmed S., Hossain M.A., Ahmed T., Ali M.S. (2021). Dietary Composition and Functional Properties of Selected Watermelon Seeds Available in Bangladesh. SAARC J. Agric..

[72] Gebru Y.A., Kim D.-W., Sbhatu D.B., Abraha H.B., Lee J.W., Choi Y.B., Kim Y.-H., Kim M.-K., Kim K.-P. (2021). Comparative analysis of total phenol, total flavonoid and in vitro antioxidant capacity of white and brown teff (*Eragrostis tef*), and identification of individual compounds using UPLC-qTOF-MS. J. Food Meas. Charact..

[73] Neglo D., Tettey C.O., Essuman E.K., Kortei N.K., Boakye A.A., Hunkpe G., Amarh F., Kwashie P., Devi W.S. (2021). Comparative antioxidant and antimicrobial activities of the peels, rind, pulp and seeds of watermelon (*Citrullus lanatus*) fruit. Sci. Afr..

