# Supplementary material for: Mineral, Nutritional, and Phytochemical Composition and Baking Properties of Teff and Watermelon Seed Flours
Source: Molecules. 2023 Apr 5;28(7):3255. doi: 10.3390/molecules28073255 (PMC10096663; doi:10.3390/molecules28073255)
Supplement: Supplementary file 1 [file molecules-28-03255-s001.zip › molecules-2300397-supplementary.pdf]

SUPPLEMENTARY MATERIALS

Mineral, Nutritional, and Phytochemical Composition and Baking Properties of Teff and Watermelon Seed Flours

Anna Jaroszewska[1], Dariusz Jedrejek[2*], Magdalena Sobolewska[1], Iwona Kowalska[2], Małgorzata Dzięcioł[3]

[1] *Department of Agroengineering, Faculty of Environmental Management and Agriculture, West Pomeranian University of Technology in Szczecin, Pawła VI Street, 71-459 Szczecin, Poland*

[2] *Department of Biochemistry and Crop Quality, Institute of Soil Science and Plant Cultivation – State Research Institute, Czartoryskich 8 Street, 24-100 Puławy, Poland*

[3] *Department of Chemical Organic Technology and Polymeric Materials, Faculty of Chemical Technology and Engineering, West Pomeranian University of Technology in Szczecin, Piastów Ave. 42, 71-065 Szczecin, Poland*

* Corresponding author

E-mail address: djedrejek@iung.pulawy.pl (D. Jedrejek)

Tel.: +48-81-4786-886

Table of Contents:

Figure S1. Demonstrative photographs of raw and processed test materials: teff seeds (A), teff flour (B), watermelon seeds (C), watermelon whole seed flour prepared in-house (D), and commercial watermelon seed pomace (defatted) flour (E) (photographs were taken by D.Jedrejek and M.Sobolewska).

Figure S2. Effect of the addition of teff and watermelon seed flours (test levels: 10%, 20% and 30% w/w) to wheat flour on the rheological properties of the dough. All results were normalized to a control sample (wheat flour type 650) = 1.0. Samples: WF – wheat flour, TF – teff flour, WSF – watermelon seed flour, DWSF – watermelon seed pomace flour.

Figure S3. UHPLC-UV-MS chromatograms of methanolic extract of teff flour. Peak numbering (outlined with circles) is according to Table 2 in the main body of the paper.

Figure S4. UHPLC-UV-MS chromatograms of methanolic extract of watermelon seed flour. Peak numbering (outlined with circles) is according to Table 2 in the main body of the paper. **Figure S5.** UHPLC-UV-MS chromatograms of methanolic extract of watermelon seed pomace flour. Peak numbering (outlined with circles) is according to Table 2 in the main body of the paper.

Figure S6. GC-MS chromatogram of methanolic extract of teff flour.

Figure S7. GC-MS chromatogram of methanolic extract of watermelon seed flour.

Figure S8. GC-MS chromatogram of methanolic extract of watermelon seed pomace flour.

Figure S9. LC-MS/MS chromatograms of melatonin (MEL) and melatonin-d_4 (MEL-d_4) standards – A), and blank sample – B).

Figure S10. LC-MS/MS chromatograms of MEL and MEL-d_4 from test samples: teff flour – A), watermelon seed flour – B), watermelon seed pomace flour – C).

Figure S11. Inhibition curves (inhibition% vs sample concentration) obtained for test samples and positive controls (Trolox or EDTA) in antiradical (ABTS and DPPH) and metal ion chelating (FCA) assays.

Table S1. The current literature data on the content of minerals as well as total protein and fat in teff and watermelon seeds and/or flours.

Table S2. Correlations between the analyzed rheological parameters.

Table S3. The current literature data on the total phenolic content and antioxidant activity of teff and watermelon seeds and/or flours.

Table S4. Calibration parameters and results of the evaluation of linearity (regression coefficient (R^2), goodness-of-fit (gof), mean ± standard deviation), the limit of detection (LOD), the limit of quantification (LOQ), reproducibility (relative standard deviation – RSD), and

recovery (mean ± standard deviation) of melatonin determination in teff, watermelon seed flour and watermelon seed pomace flour.

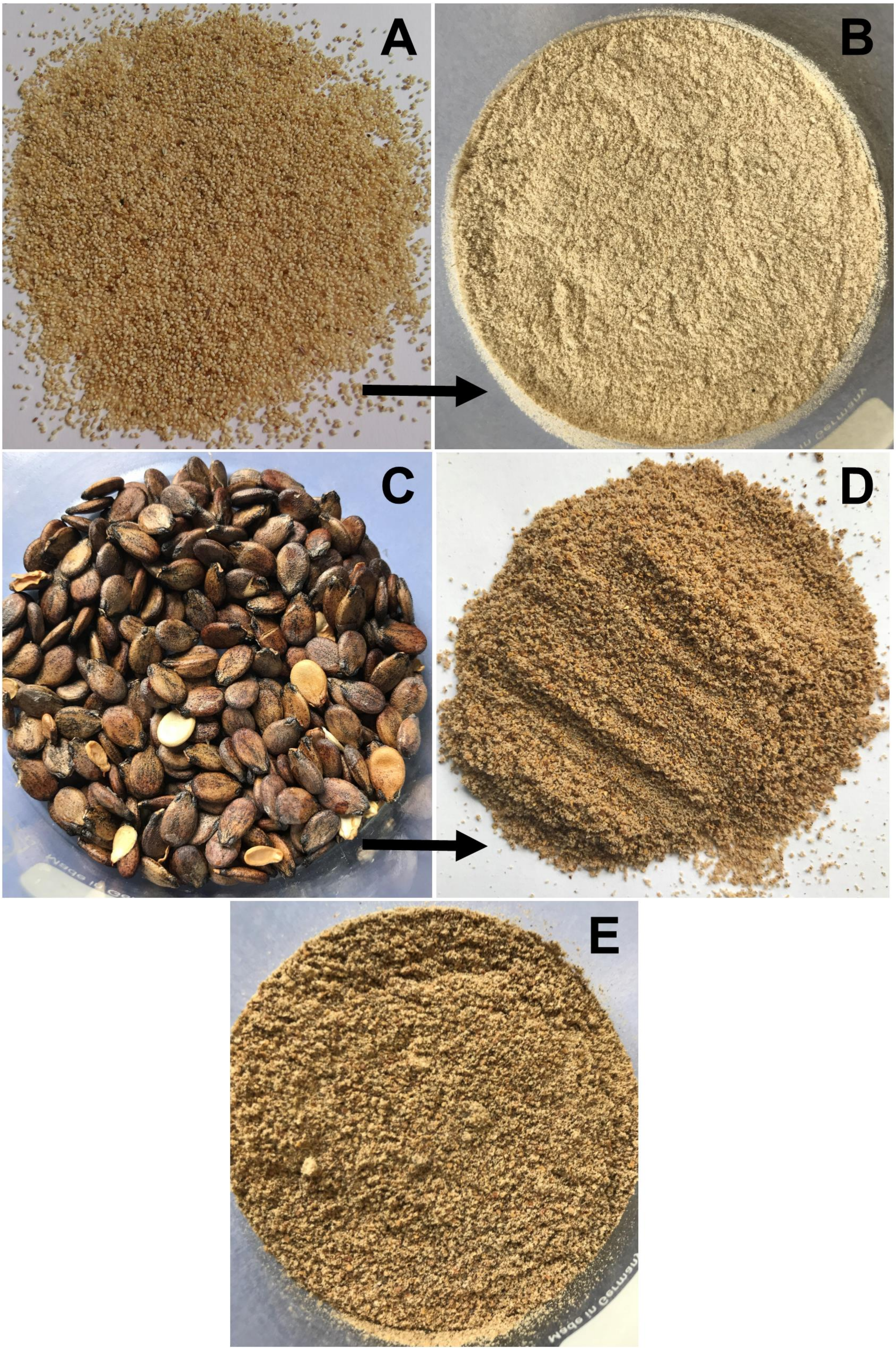

Figure S1. Demonstrative photographs of raw and processed test materials: teff seeds (**A**), teff flour (**B**), watermelon seeds (**C**), watermelon whole seed flour prepared in-house (**D**), and commercial watermelon seed pomace (defatted) flour (**E**) (photographs were taken by D.Jedrejek and M.Sobolewska).

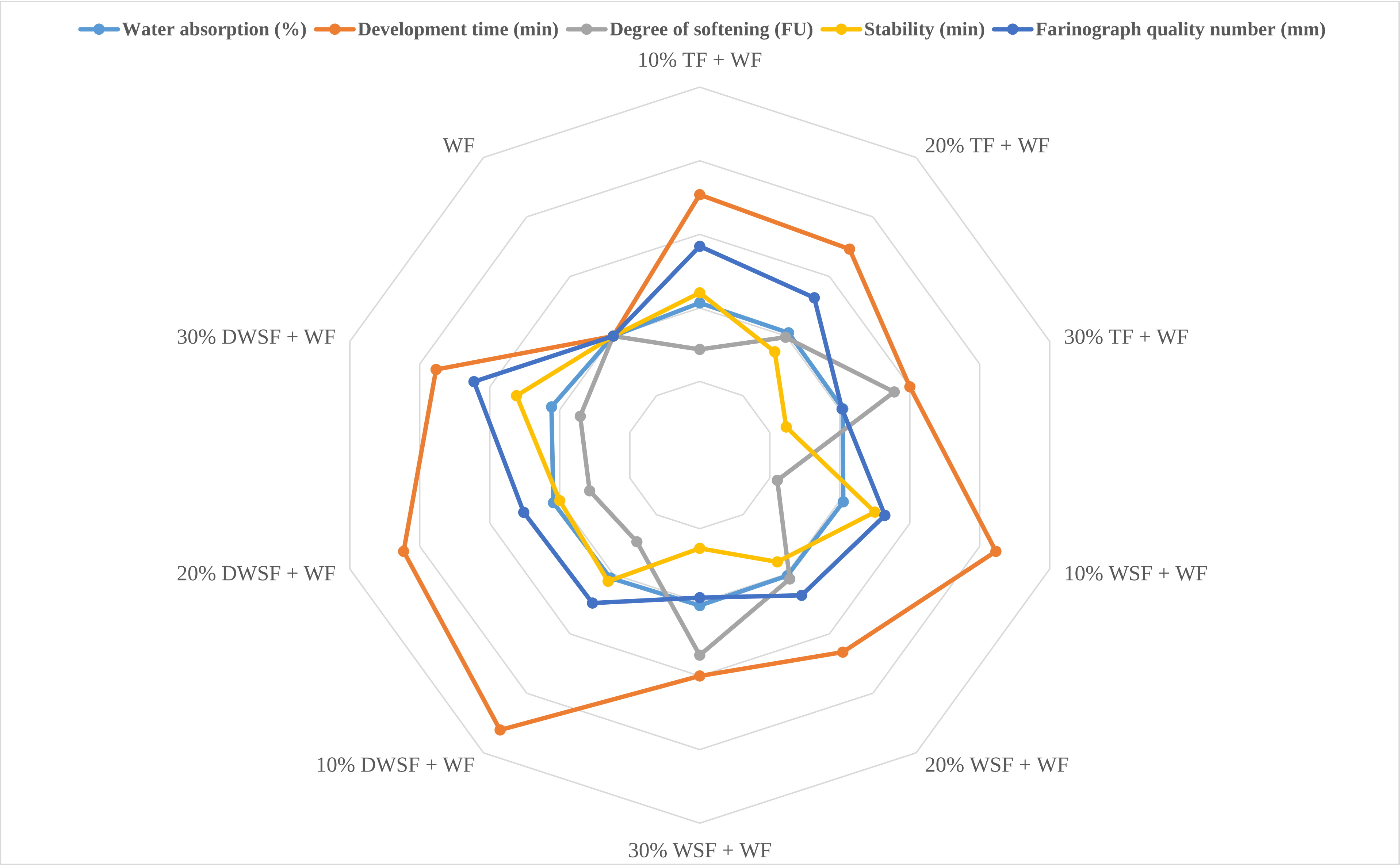

Figure S2. Effect of the addition of teff and watermelon seed flours (test levels: 10%, 20% and 30% w/w) to wheat flour on the rheological properties of the dough. All results were normalized to a control sample (wheat flour type 650) = 1.0. Samples: WF – wheat flour, TF – teff flour, WSF – watermelon seed flour, DWSF – watermelon seed pomace flour.

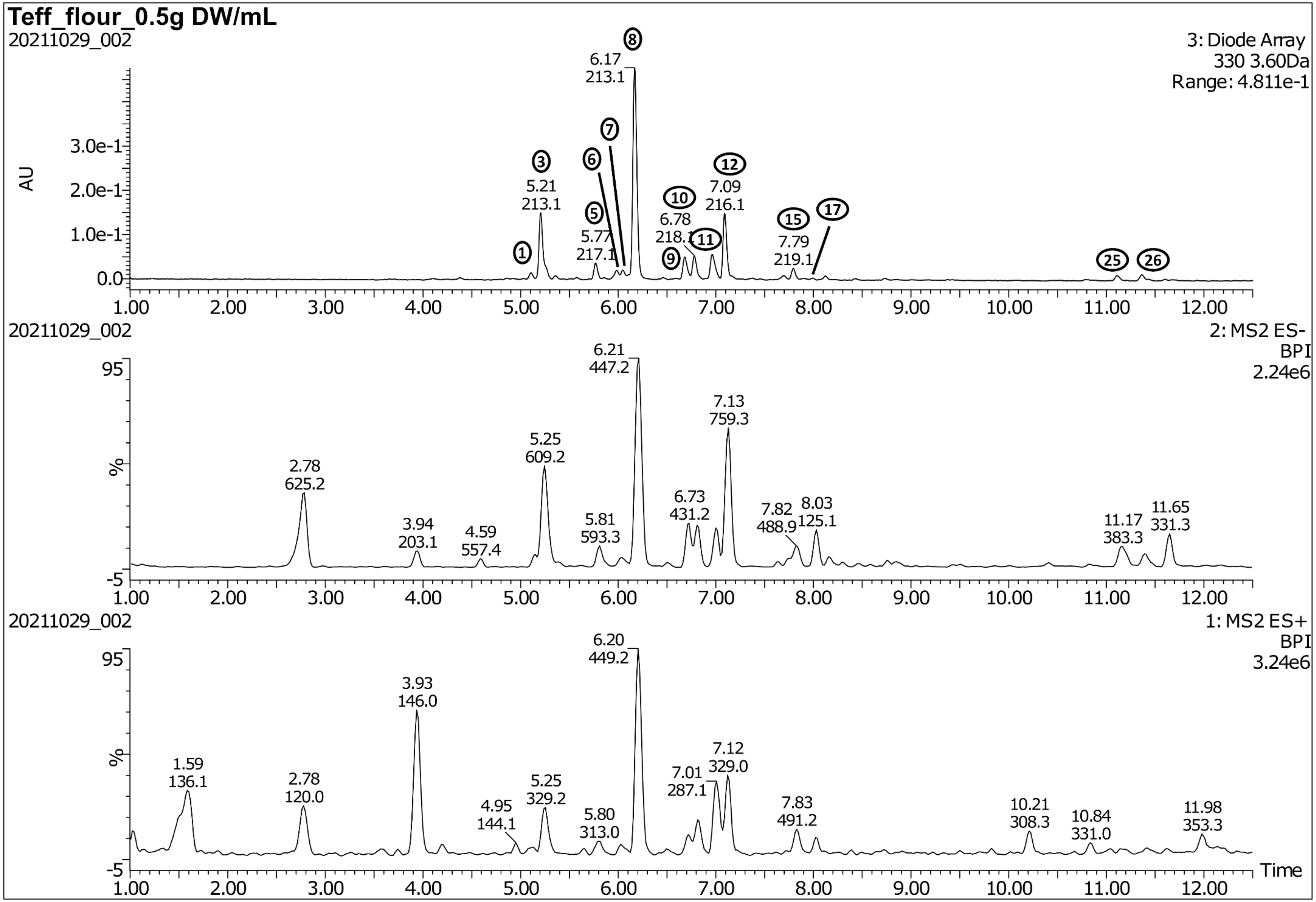

Figure S3. UHPLC-UV-MS chromatograms of methanolic extract of teff flour. Peak numbering (outlined with circles) is according to Table 2 in the main body of the paper.

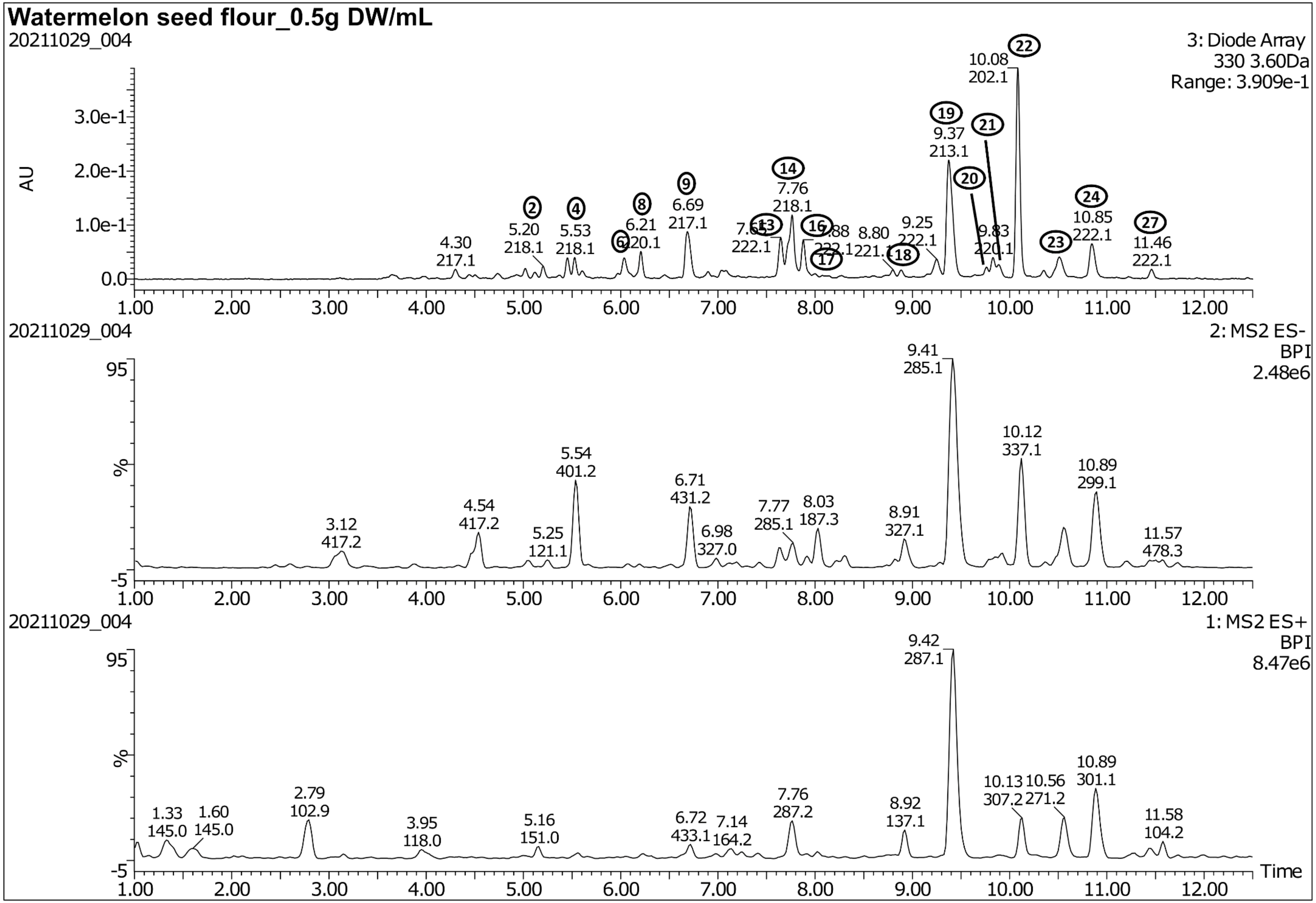

Figure S4. UHPLC-UV-MS chromatograms of methanolic extract of watermelon seed flour. Peak numbering (outlined with circles) is according to Table 2 in the main body of the paper.

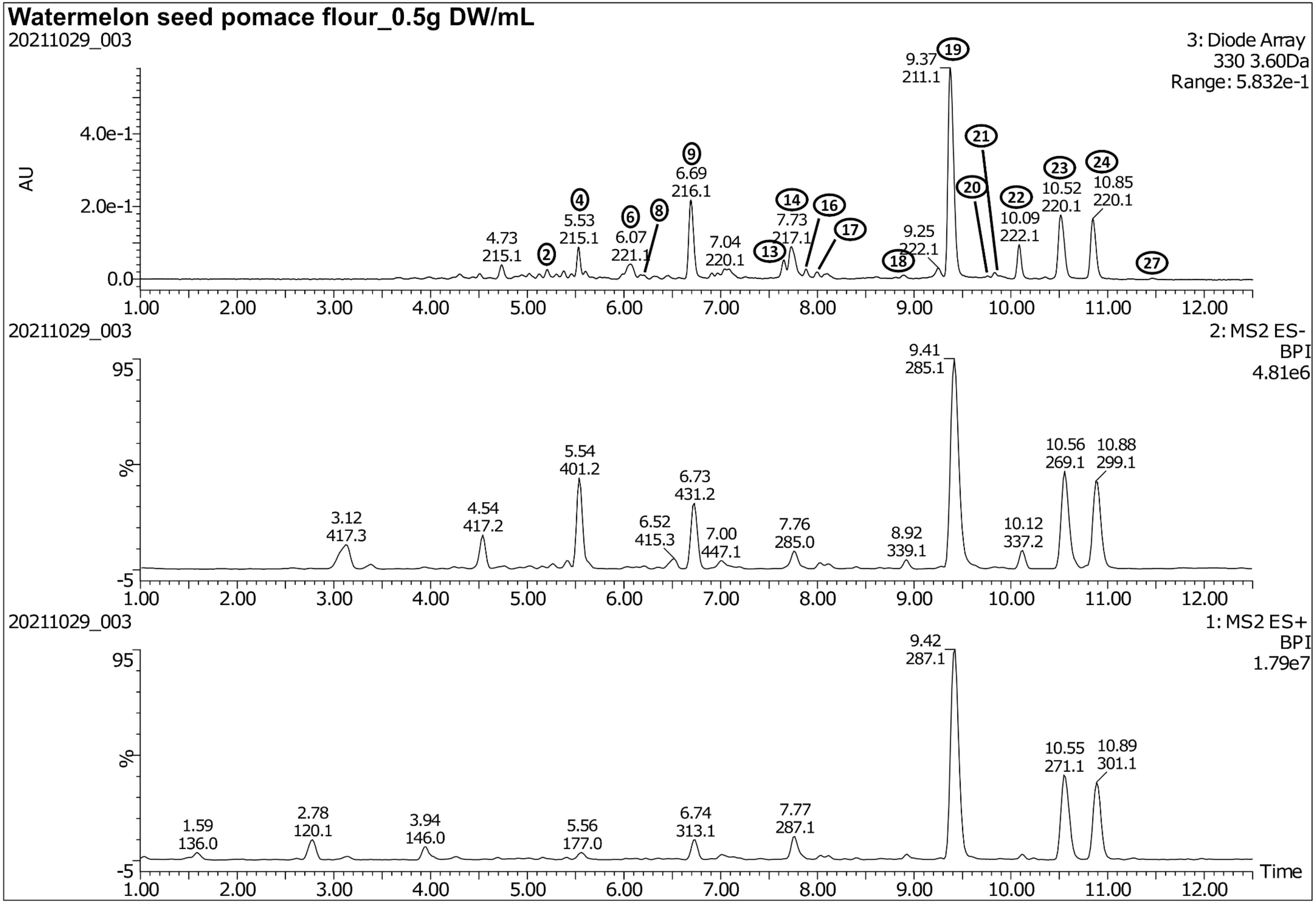

Figure S5. UHPLC-UV-MS chromatograms of methanolic extract of watermelon seed pomace flour. Peak numbering (outlined with circles) is according to Table 2 in the main body of the paper.

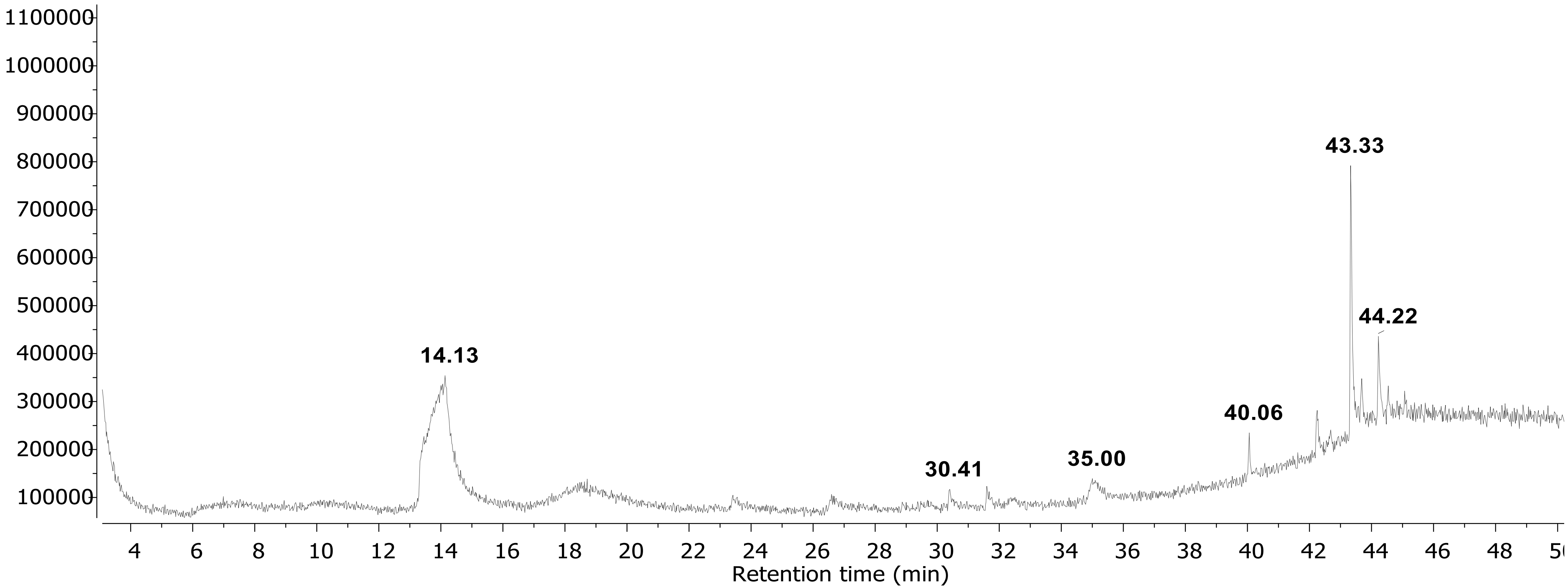

Figure S6. GC-MS chromatogram of methanolic extract of teff flour.

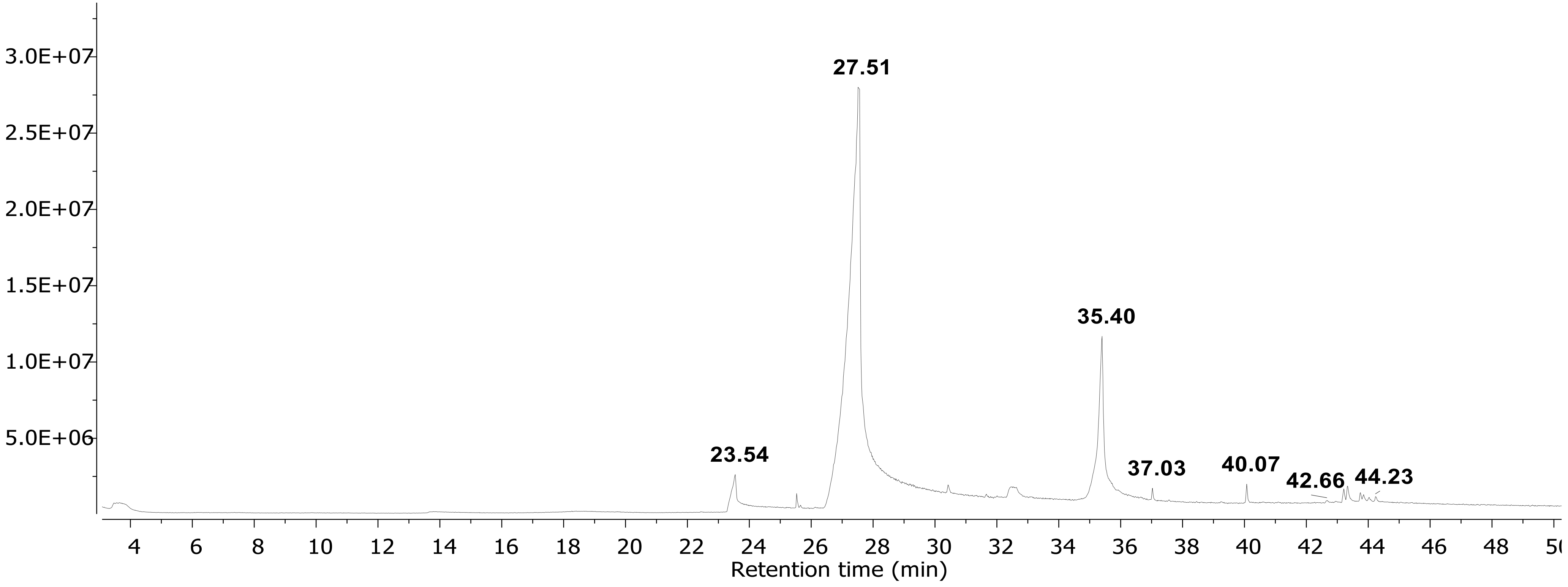

Figure S7. GC-MS chromatogram of methanolic extract of watermelon seed flour.

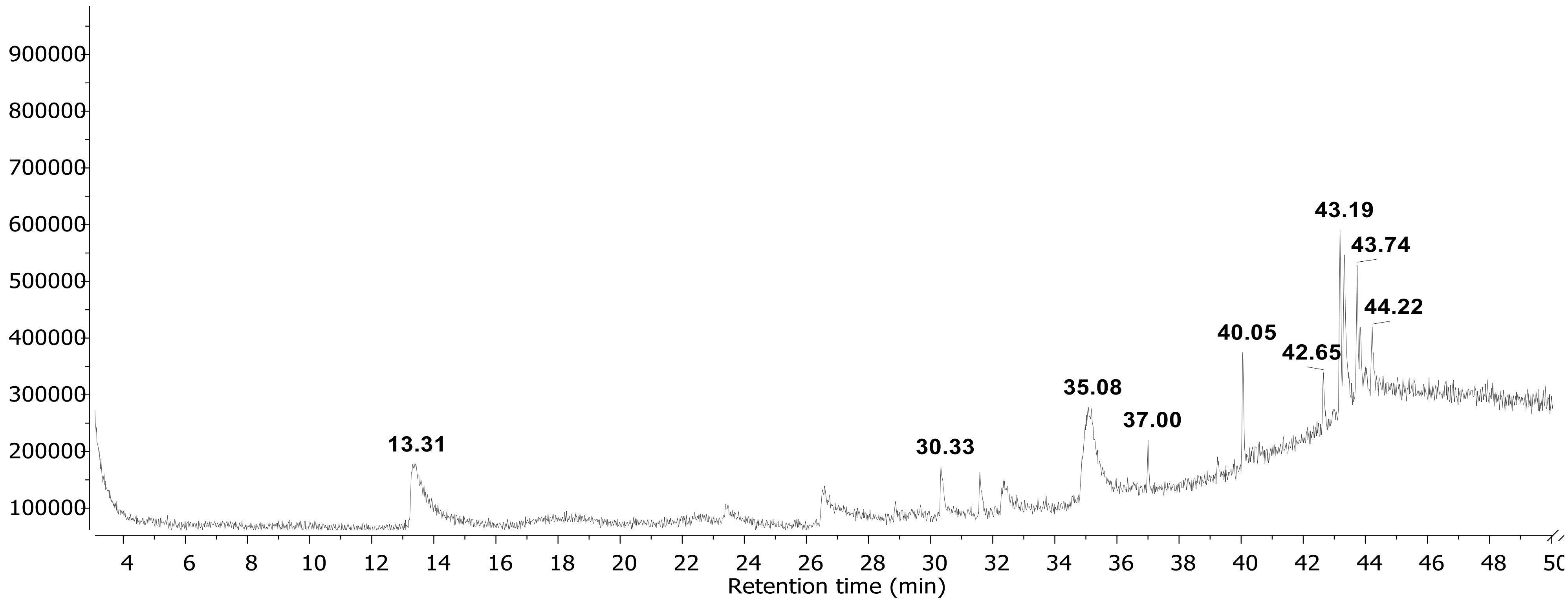

Figure S8. GC-MS chromatogram of methanolic extract of watermelon seed pomace flour.

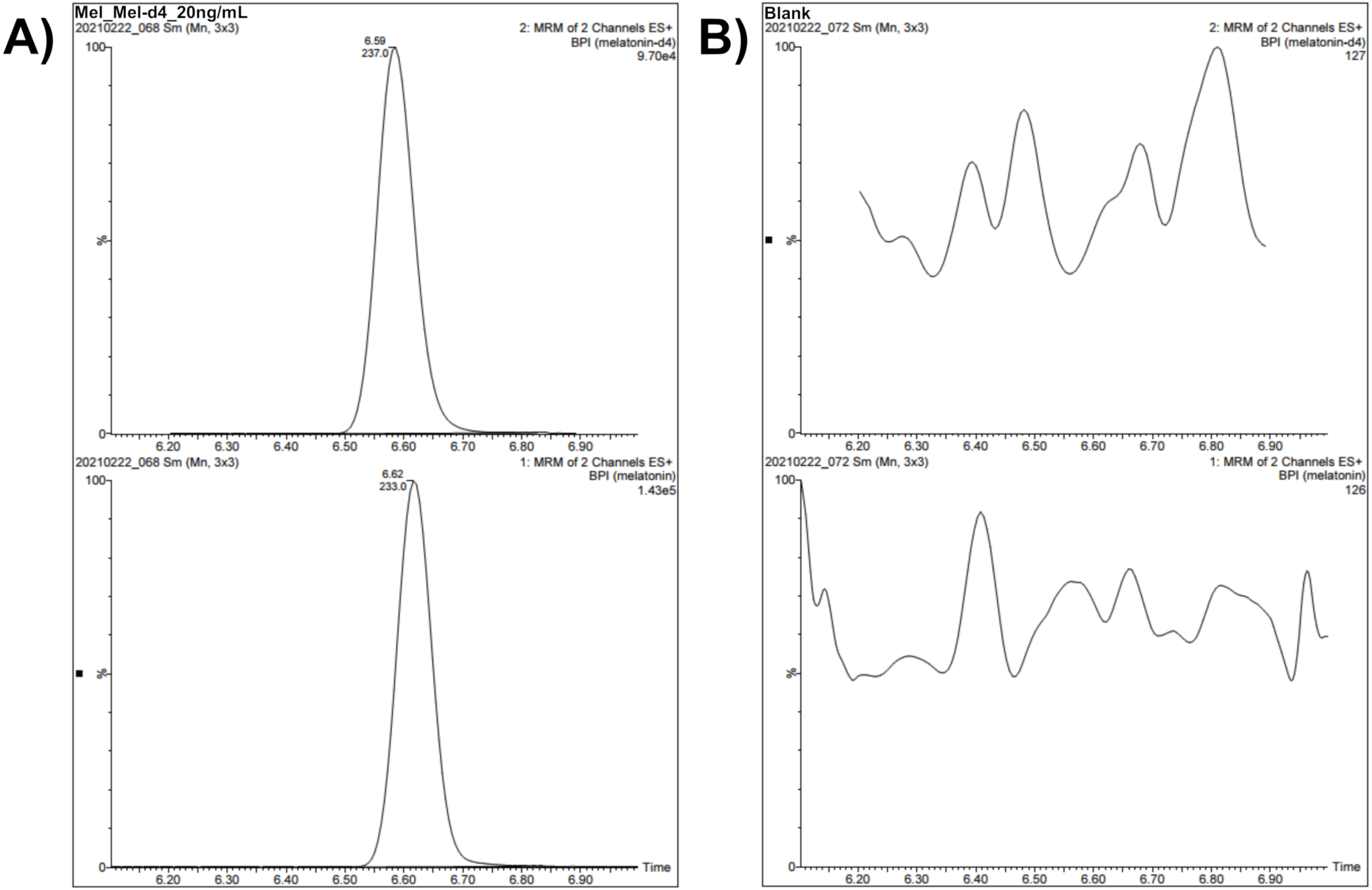

Figure S9. LC-MS/MS chromatograms of melatonin (MEL) and melatonin-*d*$_4$ (MEL-d4) standards – (**A**), and blank sample – (**B**).

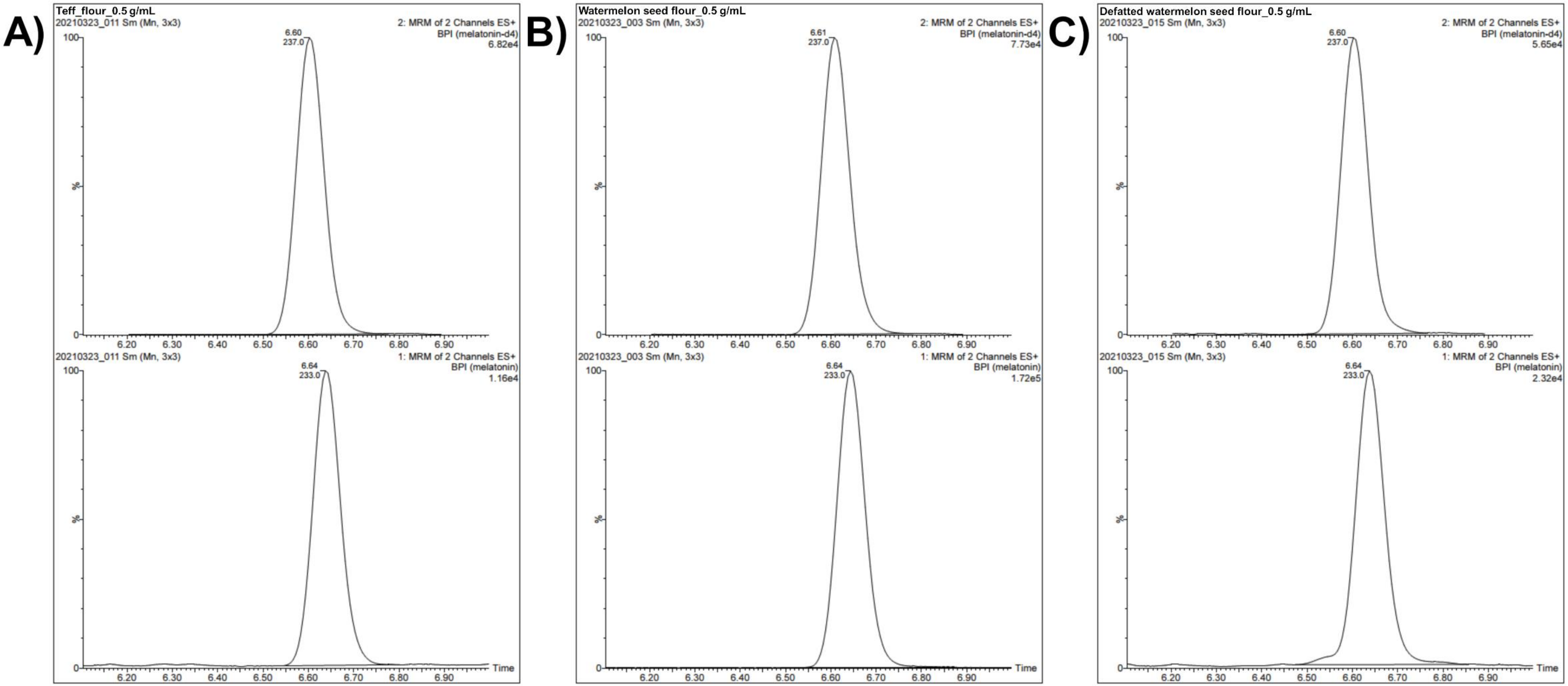

Figure S10. Chromatograms of MEL and MEL-d_4 from test samples: teff flour – (**A**), watermelon seed flour – (**B**), and watermelon seed pomace flour – (**C**).

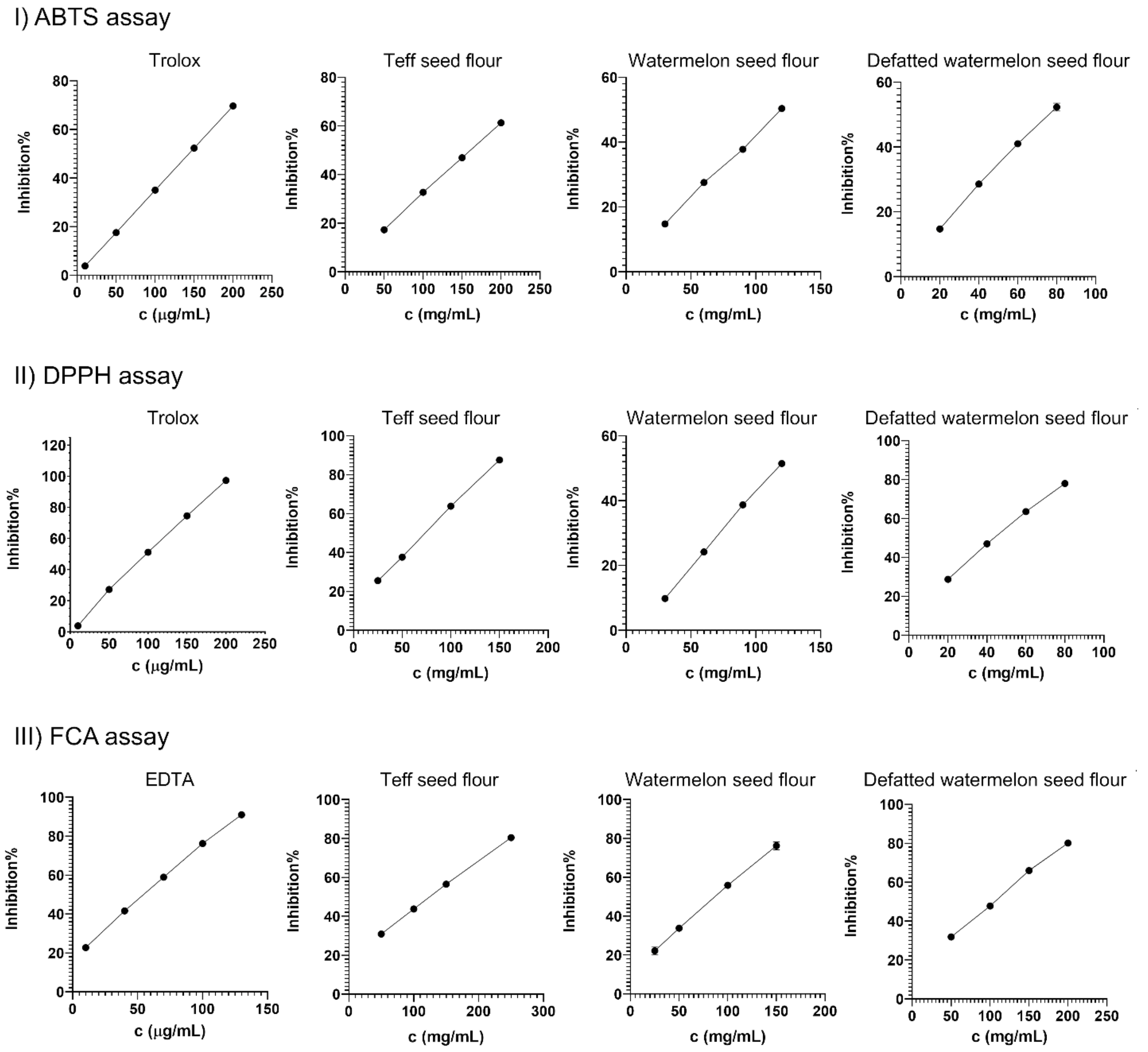

Figure S11. Inhibition curves (inhibition% vs sample concentration) of test samples and positive controls (Trolox or EDTA) in antiradical (ABTS and DPPH) and metal ion chelating (FCA) assays.

Table S1. The current literature data on the content of minerals as well as total protein and fat in teff and watermelon seeds and/or flours

No	Literature, source	Additional information	Element [mg/kg]										Total protein	Total lipids
			P	K	Ca	Mg	Na	Fe	Zn	Mn	Mo	Cu	[g/100 g, (%)]	[g/100 g, (%)]
Teff grains and flour														
1	Bultosa, G.; Taylor, J.R.N. 2004 [16].	proximate composition of teff grain	3 780-4 800	3 300-5 700	1 040-2 230	1 380-1 900	118-470	47-196	20-67	16-64	-	7.0-53.0	9.38-13.3	2.0-3.1
2	Bultosa, G. 2007 [64].	grains of 13 Ethiopian teff varietes	-	-	-	-	-	-	-	-	-	-	8.7-11.1	2.0-3.0
3	El-Alfy et al. 2012 [42].	red teff grains from Egypt and Saudi Arabia, variety unknown	-	-	-	-	-	-	-	-	-	-	20.9	4.9
4	Forsido et al. 2013 [50].	teff flour from Ethiopia, variety unknown	3 500	4 700	1 300	1 700	200	-	29.5	56.5	-	7.3	9.8	3.7
5	Baye et al. 2014 [65].	grain teff from Ethiopia, variety unknown	-	-	788.4	-	-	800.7 (unwashed), 316.1 (washed)	23.1	-	-	-	-	-
6	USDA FoodData Central [67].	seeds of 1 unknown variety of teff	4 290	4 270	1 800	1 840	120	76.3	36.3	92.4	-	8.1	13.3	2.4
7	Alemneh et al. 2022 [66].	grain teff of Dagme (DZ-Cr-438) variey from Ethiopia and whole grain teff flour from Germany (Teff-shop.de)	-	-	1 142-1 306	-	-	58-363	24.1-34.6	-	-	-	9.5-13.3	2.9-3.3
8	Our results	white teff grains from Germany (Teff-shop.de)	5 400	2 710	890	1 530	80	60.3	22.9	42.6	13.8	0.0	11.7	2.9
Watermelon seeds and flour														
1	El-Adawy, T.A.; Taha, K.M. 2001 [19].	watermelon seeds from Egypt, variety unknown	12 790	11 760	1 500	5 420	330	121	106	99		21	35.7	50.1
2	Tabiri et al. 2016 [36].	seeds of 3 watermelon varieties (Charleston gray, Crimson sweet and Black diamond) from Ghana	1 700-2 200	34 000-38 500	1 100-1 600	1 400-1 700	700-1 700	27.2-46.0	6.6-37.1	200-900	-	3.8-5.8	16.3-17.7	26.5-27.8
3	USDA FoodData Central [67].	seeds of unknown watermelon variety	7 550	6 480	540	5 150	990	72.8	102.4	16.4	-	6.86	28.3	47.4
4	Rezig et al. 2019 [68].	seeds of watermelon variety 'Ananas' from Tunisia	7 873.10	7 505	1 078.50	2 696.2	3 020	51.7	68	14	-	755.1	19.4	19.2
5	Kausar et al. 2020 [69].	watermelon seeds from Pakistan, variety unknown	-	-	-	-	-	-	-	-	-	-	26.4	46.8
6	Eke et al. 2021 [70].	watermelon seeds from Nigeria, variety unknown	1 354.2	1 880.5	704.2	1 957.5	844.6	-	-	-	-	-	19.02	43.51
7	Japu et al. 2021 [71].	seeds of 2 watermelon varieties (Charleston gray and Crimson red) from Bangladesh	-	-	-	-	-	-	-	-	-	-	22.5-27.2	30.6-36.6
8	Our results	seeds of Hungarian watermelon bought in Poland (variety unknown) and deffated watermelon seed flour (Olvita.pl)	6 050-7 500	5 140-6 310	350-500	1 540-1 720	100-440	28.7-124.2	25.2-27.5	13.8-16.1	12.5-17.5	0	20.5-25.2	8.99 (comercially defatted), 29.61

Several reports exist on the mineral content of ground teff and watermelon seeds, however, there is large element variation in presented results, an example of which is a broad iron range in Eragrostis tef seeds (47-363 mg/kg). The reasons for a large mineral data discrepancies in grain and flour are not entirely clear, but they may be influenced by genetics (different landraces or cultivars), climate and soil factors as well as those related to material processing (sieving and milling) and laboratory element analysis methodology.

Table S2

Correlations between the analyzed rheological parameters.

Farinograph parameter	Observed correlation coefficients				
	Water absorption	Development time	Stability time	Degree of softening	Farinograph quality number
Water absorption	-	0.67	0.48	-0.38	0.79
Development time	0.67	-	0.47	-0.65	0.58
Stability time	0.48	0.47	-	-0.89	0.80
Degree of softening	-0.38	-0.65	-0.89	-	-0.68
Farinograph quality number	0.79	0.58	0.80	-0.68	-
positive correlation →	high	moderate			
negative correlation →	high	moderate			

Table S3. The current literature data on the total phenolic content and antioxidant activity of teff and watermelon seeds and/or flours

No	Literature, source	Additional information	Total phenolic content (TPC) [mg GAE/g]* or [mg CE/g]#	Antioxidant activity			
				ABTS method	DPPH method	FRAP method	ORAC method
Teff seeds and flour							
1	Gebru et al. 2021 [72].	grains of 2 white teff varieties (Addis and Mekelle) from Ethiopia, extracts of soluble polyphenols prepared with 80% aqueous MeOH	1.03-1.84 *	-	3.65-5.24 µM TEAC/g	5.25-7.0 µM TEAC/g	-
2	Forsido et al. 2013 [50].	teff flour from Ethiopia (variety unknown), methanolic extract prepared by sonication	1.24 *	-	IC_{50}, 22.35 mg/mL	1.76 µM TE/g	-
3	Shumoy, H.; Raes, K. 2016 [31].	grains of 7 teff varieties (Boset, Dega, Quncho, Simada, Tsedey, Zagurey and Zezew) from Ethiopia, extracts of soluble polyphenols prepared with 80% aqueous MeOH using homogenizer	0.37-0.71 *	-	2.9-6.5 µM TE/g	6.2-15.7 µM FE^{2+}/g	-
4	Kotaskova et al. 2016 [32].	grains of white teff from Bolivia and USA (varieties unknown), extracts of free phenolics prepared with 80% aqueous MeOH by sonication	0.9-1.0 *	1.7-2.3 µM TEAC/g	1.4-1.6 µM TEAC/g	-	-
5	Salawu et al., 2013 [30].	grains of white teff from South Africa (variety unknown), extract prepared with MeOH acidified with 1% HCl by shaking	≈ 7.0 #	≈ 40 µM TE/g	-	≈ 1.1 mg vitC Eq/g	≈ 600 µM TE/g
Watermelon seeds and flour							
1	Neglo et al., 2021 [73].	watermelon seeds from Ghana (Charleston Gray variety), methanolic extract prepared by shaking at room temperature	0.042 *	inhibition, 56.1%	inhibition, 41.1%	-	-
2	Fadimu et al. 2020 [37].	watermelon seeds from Yemen (variety unknown), extract prepared with aqueous EtOH by sonication	32.15 *	-	inhibition, 85.9%	-	-
3	Tabiri et al. 2016 [36]	seeds of 3 watermelon varieties (Charleston Gray, Crimson Sweet and Black Diamond) from Ghana, methanolic extract prepared by sonication	14.9-54.2 *	-	82.6-130.3 µM TE/g	-	-

* Results expressed as [mg GAE/g]; # Results expressed as [mg CE/g]

Table S4

Calibration parameters and results of the evaluation of linearity (regression coefficient (R^2), goodness-of-fit (gof), mean ± standard deviation), the limit of detection (LOD), the limit of quantification (LOQ), reproducibility (relative standard deviation – RSD), and recovery (mean ± standard deviation) of melatonin determination in teff, watermelon seed flour and watermelon seed pomace flour.

Analyte / sample	Calibration range (ng/mL)	R^2	gof (%) (*n*=5)[a]	LOD (ng/mL)[b]	LOQ (ng/mL)[c]	RSD (%) intraday (*n*=5) / interday (*n*=10)[d]	Recovery (%)[e]
Melatonin standard	1.0–50.0	0.9996	6.98 ± 2.60	0.04	0.12		
Teff flour						4.89/5.28	72.33 ± 4.80
Watermelon seed flour						6.13/6.58	49.01 ± 2.48
Watermelon seed pomace flour						1.14/1.61	79.76 ± 2.14

[a] – gof (%) was calculated according to the formula $g = \sqrt{\Sigma(\%\ \text{deviation})^2/(n-1)}$, with %deviation = ($x_{\text{calculated conc}}$ – $x_{\text{nominal value}}$/$x_{\text{nominal value}}$) × 100. The gof (%) should fall within the range ≤ 10%.

[b] – LOD (ng/mL) was determined as MEL concentration with a signal-to-noise (S/N) ratio of ≥ 3.

[c] – LOQ (ng/mL) was determined as 3 × LOD concentration of MEL.

[d] – RSD (%) was calculated from MEL response (peak area) in test samples analyzed five times during the day (intraday) and 10 times over two days (interday).

[e] – Recovery (%) was estimated based on the behavior of internal standard (MEL-d_4), by comparing the response (peak area) of standard and spiked (20 ng/mL) test samples.
